# Association of Damaging Variants in Genes With Increased Cancer Risk Among Patients With Congenital Heart Disease

**DOI:** 10.1001/jamacardio.2020.4947

**Published:** 2020-10-21

**Authors:** Sarah U. Morton, Akiko Shimamura, Peter E. Newburger, Alexander R. Opotowsky, Daniel Quiat, Alexandre C. Pereira, Sheng Chih Jin, Michelle Gurvitz, Martina Brueckner, Wendy K. Chung, Yufeng Shen, Daniel Bernstein, Bruce D. Gelb, Alessandro Giardini, Elizabeth Goldmuntz, Richard W. Kim, Richard P. Lifton, George A. Porter, Deepak Srivastava, Martin Tristani-Firouzi, Jane W. Newburger, J. G. Seidman, Christine E. Seidman

**Affiliations:** 1 Division of Newborn Medicine, Department of Medicine, Boston Children’s Hospital, Boston, Massachusetts; 2 Department of Pediatrics, Harvard Medical School, Boston, Massachusetts; 3 Department of Pediatric Hematology/Oncology, Boston Children’s Hospital, Boston, Massachusetts; 4 Dana Farber Cancer Institute, Boston, Massachusetts; 5 Department of Pediatrics University of Massachusetts Medical School, Worcester; 6 Molecular, Cell, and Cancer Biology, University of Massachusetts Medical School, Worcester; 7 Department of Cardiology, Boston Children’s Hospital, Boston, Massachusetts; 8 Cardiovascular Division, Brigham and Women’s Hospital, Boston, Massachusetts; 9 Department of Pediatrics, Harvard Medical School, Boston, Massachusetts; 10 Department of Genetics, Harvard Medical School, Boston, Massachusetts; 11 Department of Genetics, Yale University School of Medicine, New Haven, Connecticut; 12 Department of Pediatrics, Yale University School of Medicine, New Haven, Connecticut; 13 Department of Pediatrics, Columbia University Medical Center, New York, New York; 14 Department of Medicine, Columbia University Medical Center, New York, New York; 15 Departments of Systems Biology, Columbia University Medical Center, New York, New York; 16 Departments of Biomedical Informatics, Columbia University Medical Center, New York, New York; 17 Department of Pediatrics, Cardiology, Stanford University, Stanford, California; 18 Mindich Child Health and Development Institute and Department of Pediatrics, Icahn School of Medicine at Mount Sinai, New York, New York; 19 Cardiorespiratory Unit, Great Ormond Street Hospital, London, UK; 20 Division of Cardiology, Children’s Hospital of Philadelphia, Department of Pediatrics, Perelman School of Medicine, University of Pennsylvania, Philadelphia; 21 Pediatric Cardiac Surgery, Children's Hospital of Los Angeles, Los Angeles, California; 22 Laboratory of Human Genetics and Genomics, The Rockefeller University, New York, New York; 23 Department of Pediatrics, University of Rochester Medical Center, The School of Medicine and Dentistry, Rochester, New York; 24 Gladstone Institute of Cardiovascular Disease, San Francisco, California; 25 Division of Pediatric Cardiology, University of Utah, Salt Lake City; 26 Howard Hughes Medical Institute, Chevy Chase, Maryland

## Abstract

**Importance:**

Patients with congenital heart disease (CHD), the most common birth defect, have increased risks for cancer. Identification of the variables that contribute to cancer risk is essential for recognizing patients with CHD who warrant longitudinal surveillance and early interventions.

**Objective:**

To compare the frequency of damaging variants in cancer risk genes among patients with CHD and control participants and identify associated clinical variables in patients with CHD who have cancer risk variants.

**Design, Setting, and Participants:**

This multicenter case-control study included participants with CHD who had previously been recruited to the Pediatric Cardiac Genomics Consortium based on presence of structural cardiac anomaly without genetic diagnosis at the time of enrollment. Permission to use published sequencing data from unaffected adult participants was obtained from 2 parent studies. Data were collected for this study from December 2010 to April 2019.

**Exposures:**

Presence of rare (allele frequency, <1 × 10^−5^) loss-of-function (LoF) variants in cancer risk genes.

**Main Outcomes and Measures:**

Frequency of LoF variants in cancer risk genes (defined in the Catalogue of Somatic Mutations in Cancer–Cancer Gene Consensus database), were statistically assessed by binomial tests in patients with CHD and control participants.

**Results:**

A total of 4443 individuals with CHD (mean [range] age, 13.0 [0-84] years; 2225 of 3771 with reported sex [59.0%] male) and 9808 control participants (mean [range] age, 52.1 [1-92] years; 4967 of 9808 [50.6%] male) were included. The frequency of LoF variants in regulatory cancer risk genes was significantly higher in patients with CHD than control participants (143 of 4443 [3.2%] vs 166 of 9808 [1.7%]; odds ratio [OR], 1.93 [95% CI, 1.54-2.42]; *P* = 1.38 × 10^−12^), and among CHD genes previously associated with cancer risk (58 of 4443 [1.3%] vs 18 of 9808 [0.18%]; OR, 7.2 [95% CI, 4.2-12.2]; *P* < 2.2 × 10^−16^). The LoF variants were also nominally increased in 14 constrained cancer risk genes with high expression in the developing heart. Seven of these genes (*ARHGEF12*, *CTNNB1*, *LPP*, *MLLT4*, *PTEN*, *TCF12*, and *TFRC*) harbored LoF variants in multiple patients with unexplained CHD. The highest rates for LoF variants in cancer risk genes occurred in patients with CHD and extracardiac anomalies (248 of 1482 individuals [16.7%]; control: 1099 of 9808 individuals [11.2%]; OR, 1.59 [95% CI, 1.37-1.85]; *P* = 1.3 × 10^−10^) and/or neurodevelopmental delay (209 of 1393 individuals [15.0%]; control: 1099 of 9808 individuals [11.2%]; OR, 1.40 [95% CI, 1.19-1.64]; *P* = 9.6 × 10^−6^).

**Conclusions and Relevance:**

Genotypes of CHD may account for increased cancer risks. In this cohort, damaging variants were prominent in the 216 genes that predominantly encode regulatory proteins. Consistent with their fundamental developmental functions, patients with CHD and damaging variants in these genes often had extracardiac manifestations. These data may also implicate cancer risk genes that are repeatedly varied in patients with unexplained CHD as CHD genes.

## Key Points

**Question:**

Do damaging gene variants account for increased cancer risk in patients with congenital heart disease (CHD)?

**Findings:**

In this case-control study, loss-of-function variants in cancer risk genes were increased approximately 1.3-fold in 4443 patients with CHD compared with 9808 control participants. This burden was highest in cancer risk genes previously associated with CHD (7.2-fold) or that regulate gene expression (1.9-fold); patients with CHD and extracardiac anomalies and/or neurodevelopmental delay had the highest rates of damaging variants in cancer risk genes.

**Meaning:**

Genetic analyses of patients with CHD may identify precise causes of heart malformations and also patients with CHD and increased cancer risks.

## Teaser

This case-control study compares the frequency of damaging cancer risk gene variants in patients with congenital heart disease vs control participants and identifies associated clinical variables.

## Introduction

The growing population of adults with congenital heart disease (CHD)^1^ has created increased recognition of additional health issues, including a 1.4-fold to 2-fold higher cancer prevalence than in the general population.^2,3,4^ While radiation exposure from therapeutic interventions can increase cancer risk (CR),^5^ the diversity of malignant conditions outside of radiation fields suggests other risk factors.^3^ Additional risks and mechanisms that link CHD to cancer are unknown.

Damaging gene variants contribute to both CHD^6,7^ and cancer,^8^ hinting that these disorders share molecular relationships. This model is supported by the increased prevalence of damaging variants in CR genes among children with developmental delays,^9^ including autism and intellectual disabilities, which occur in some patients with CHD. To explore potential molecular relationships, we analyzed rare loss-of-function (LoF) variants in CR genes among a large CHD cohort and defined accompanying clinical features. These analyses identify patients with CHD and the highest CR gene burden, who may warrant longitudinal cancer screening.

## Methods

### Study Participants and Ethical Approval

The multicenter case-control study was reviewed and approved by the relevant institutional review boards, including at Boston Children’s Hospital. Written informed consent was obtained at the time of enrollment. We studied participants in the Pediatrics Cardiovascular Genetics Consortium^7^ with undefined causes for CHD at the time of enrollment and unaffected control participants in studies of autism^7^ and schizophrenia^10^ (eTable 1 in the Supplement).

### CR Genes

The Catalogue of Somatic Mutations in Cancer–Cancer Gene Consensus database defines 723 CR genes (eTable 2 in the Supplement).^8^ This includes 38 CR genes that also cause CHD (eTable 3 in the Supplement and Online Mendelian Inheritance in Man [OMIM]; https://omim.org/), 216 CR genes that regulate RNA transcription or processing, and 227 CR genes with LoF mechanisms, including 107 genes with LoF germline mechanisms.

### Variant Calls and Statistical Analyses

Whole-exome sequences from patients with CHD and control participants were processed using established pipelines^7^ to identify rare (allele frequency, ≤1 × 10^−5^) heterozygous LoF variants. All *P* values reflect binomial tests after Bonferroni correction with a *P* value threshold of 1.67 × 10^−3^ (10 gene lists and 3 comparisons). A false discovery rate *P* < .05 was used as the significant threshold throughout. The eMethods in the Supplement includes further methodological details. Data were collected for this study from December 2010 to April 2019. The software program R version 3.6.0 (R Foundation for Statistical Computing) was used for analysis.

## Results

### LoF Variants in CR Genes Among Patients With CHD

Initial analyses of LoF variants in CR genes (eTable 4 and eMethods in the Supplement) and prespecified subsets (Table 1) demonstrated significantly higher frequencies in patients with CHD who were randomly assigned to the discovery group (n = 2222) or the replication group (n = 2221) in comparisons with independent control cohorts (n = 3578 and n = 6230; eTables 5 and 6 in the Supplement). As such, we present combined data from 4443 patients with CHD and 9808 control participants (Table 1). Patients with CHD ranged in age from 0 to 84 years, with a mean (SD) age of 13.0 (14.8) years; 2225 of 3771 patients with CHD with a reported sex (59.0%) were male. Control participants ranged in age from 21 to 92 years, with a mean (SD) age of 52.1 years, and 4967 of 9808 participants (50.6%) were male.

**Table 1.** Patients With Congenital Heart Disease (CHD) and Rare Loss-of-Function (LoF) Variants in Subsets of the Catalogue of Somatic Mutations in Cancer–Cancer Gene Consensus Cancer Risk Genes

Genes	No.	Patients with CHD	Control participants	Odds ratio (95% CI)	Binomial *P *value^a^
Total participants	14 251	4443	9808	NA	NA
All cancer risk	723	642	1099	1.34 (1.21-1.49)	2.31 × 10^−11^^b^
OMIM CHD^c^	38	68	18	7.19 (4.23-12.22)	<2.2 × 10^−16^^b^
Regulatory	216	143	166	1.93 (1.54-2.42)	1.38 × 10^−12^^b^
Regulatory and OMIM CHD^c^	17	40	9	9.89 (4.80-20.40)	<2.2 × 10^−16^^b^
Regulatory without OMIM CHD^c^	199	103	157	1.46 (1.13-1.88)	2.01 × 10^−4^
Non-OMIM CHD	685	585	1082	1.22 (1.10-1.36)	5.245 × 10^−6^
Nonregulatory	507	516	942	1.24 (1.10-1.39)	5.46 × 10^−6^
LoF cancer mechanism	227	240	376	1.43 (1.21-1.69)	1.58 × 10^−7^
Recessive LoF^d^	135	158	274	1.28 (1.05-1.57)	1.68 × 10^−3^
Dominant LoF^d^	46	53	50	2.36 (1.60-3.47)	3.45 × 10^−8^^b^
Non-LoF cancer mechanism	496	422	751	1.27 (1.12-1.43)	4.46 × 10^−6^

Abbreviations: NA, not applicable; OMIM, Online Mendelian Inheritance in Man.

^a^ Bonferroni significance *P* value threshold: 1.67 × 10^−3^ (10 gene lists by 3 comparisons).

^b^ Significant in both subanalyses.

^c^ The OMIM CHD genes with dominant patterns of transmission.

^d^ Five genes have both dominant and recessive cancer variants; 51 are not characterized.

The presence of CR variants was not associated with any specific CHD subtype (eTable 7 in the Supplement). Most patients with CHD (599 of 642 [93.3%]) had a single CR variant, while a minority had 2 CR vaiants (43 of 642 [6.7%]) or 3 CR variants (3 of 642 [0.5%]). No individual had 2 independent CR variants in the same gene. Analyses restricted to participants of European ancestry (CHD: 448 of 3106 individuals [14.4%]; controls: 849 of 9501 individuals [8.9%]) remained significant (odds ratio [OR], 1.72 [95% CI, 1.52-1.94]; *P* < 2.2 × 10^−16^; eTable 8 in the Supplement). Because many CR genes are associated with adult-onset malignant conditions, we compared LoF variant frequencies in patients with CHD who were younger than 16 years (n = 3338) or older than 16 years (n = 1105). A higher proportion of older individuals had LoF variants, albeit with comparable ORs (1.33-1.37; eTable 9 in the Supplement).

Thirty-eight CR genes have dominant patterns of transmission for CHD (denoted OMIM; Table 1); these genes had significantly more LoF variants in patients with CHD than control participants (OR, 7.19 [95% CI, 4.23-12.22]; *P* < 2.2 × 10^−16^). The presence of LoF variants was also increased among CR genes with regulatory functions (OR, 1.93 [95% CI, 1.54-2.42]; *P* = 1.38 × 10^−12^), a prominent feature of many CR and CHD genes. Genes found in both categories (OMIM and regulatory; n = 17) had the highest frequency of LoF variants in individuals with CHD (OR, 9.89 [95% CI, 4.80-20.40]; *P* < 2.2 × 10^−16^). The CR genes with dominant variants that cause cancer by haploinsufficiency (n = 46) were also enriched in those with CHD (OR, 2.36 [95% CI, 1.60-3.47]; *P* = 3.45 × 10^−8^; Table 1).

### Comorbidities in Patients With CHD and LoF Variants in CR Genes

Damaging CR variants are increased in individuals with developmental delays.^9^ Because these delays can occur with CHD, we partitioned patients into those with extracardiac anomalies (ECA; n = 1482), neurodevelopmental defects (NDD; n = 1393), both ECA and NDD (n = 878), and neither ECA or NDD (isolated CHD; n = 1379). The LoF variants in CR genes were highest among patients with CHD and ECA (CHD: 248 of 1482 individuals [16.7%]; control: 1099 of 9808 individuals [11.2%]; OR, 1.59 [95% CI, 1.37-1.85]; *P* = 1.3 × 10^−10^; eTable 9 in the Supplement) and patients with CHD and NDD (CHD: 209 of 1393 individuals [15.0%]; control: 1099 of 9808 individuals [11.2%]; OR, 1.40 [95% CI, 1.19-1.64]; *P* = 9.6 × 10^−6^), while the LoF in CR genes in patients with isolated CHD was comparable with that of control participants. Notably, 10 genes had LoF variants in 3 or more patients with CHD and NDD or CHD and ECA (supporting cohort size for statistics: CHD: 1482 individuals with ECA and 1393 with NDD; control: 9808 individuals; *P* < .05), including 2 CR regulatory genes (catenin beta 1 [*CTNNB1*; in 3 participants with CHD and ECA, 2 with CHD and NDD, and 0 control participants) and transcription factor 12 [*TCF12*; in 3 patients with CHD and NDD, 2 patients with CHD and ECA, and 2 control participants]; eTable 10 in the Supplement). However, we observed no significant functional enrichment in genes with LoF variants within each CHD group (eMethods in the Supplement).

### CR Genes as Candidate CHD Genes

Given the dual role of some genes in both CHD and cancer, we considered if some CR genes might contribute to CHD. When considering only patients with CHD without pathogenic variants in OMIM CHD genes (n = 4293), we identified significantly more LoF variants in affected individuals (576 in 4293 [13.4%]) than control participants (1080 of 9744 [11.1%]; *P* = 1.2 × 10^−6^; OR, 1.24 [95% CI, 1.12-1.39]; eTable 9 in the Supplement). Moreover, 7 CR genes that are LoF intolerant (probability of LoF intolerance by the Genome Aggregation Database, >0.50) and highly expressed in the developing heart had LoF variants in 3 or more patients with unexplained CHD (rho guanine nucleotide exchange factor 12 [*ARHGEF12*], *CTNNB1*, lipoma-preferred partner [*LPP*], afadin [*MLLT4*], phosphatase and tensin homolog [*PTEN*], *TCF12*, and transferrin receptor [*TFRC*] genes; Table 2).

**Table 2.** Seven Cancer Risk Genes With Multiple Loss-of-Function (LoF) Variants in Patients With Unexplained Congenital Heart Disease (CHD)

Gene	CHD LoF	Non-CHD LoF	pLI	Heart expression rank	Human CHD gene	CR regulatory gene	Known gene syndrome/phenotype	No. of patients with CHD and CHD and ECA phenotype data	CHD phenotypes (No. of patients)	No. of patients with CHD and ECA phenotypes	Proportion of patients with CHD and NDD, No./total No.
*KDR*	7	0	0.98	87	No	No	Hemangioma	3	LVOTO (1) and ToF (2)	1 With microcephaly, micrognathia, inguinal hernia, cryptorchidism, and hydrocephalus	1/1
*TCF12*	5	2	0.97	89	No	Yes	Craniosynostosis	5	ASD (1), Ebstein anomaly (1), and LVOTO (3)	2 With bitemporal narrowing (1), abdominal heterotaxy (1), absent corpus callosum and seizure disorder (1), and/or neonatal AML (1)	3/3
*CTNNB1*	3	0	1	99	No	Yes	Neurodevelopmental disorder	3	ASD (1), dilated tricuspid valve (1), and ToF(1)	3 With microcephaly (2), strabismus (2), astigmatism (1), micrognathia (1), congenital scoliosis (1), and/or hypotonia (2)	2/2
*ARHGEF12*	3	0	1	91	No	No	None	3	ASD (2) and truncus arteriosus (1)	1 With hearing loss	3/3
*MLLT10*	3	0	1	85	No	Yes	None	2	ASD or VSD (1) and LVOTO (1)	None with ECA	1/1
*PTEN*	3	1	0.98	78	No	No	Cowden	3	ASD (2) and VSD (1)	2 With macrocephaly (2), airway malacia (1), congenital scoliosis and abnormal vertebrae (1), Chiari malformation and hydrocephalus (1), and/or Cowden features (1)	2/2
*TFRC*	3	1	0.78	82	No	No	Immunodeficiency	3	Pulmonary atresia/stenosis (2) and ToF (1)	2 With frontal bossing (1), and/or intestinal atresia and VATER association (1)	1/1

Abbreviations: AML, acute myelogenous leukemia; *ARHGEF12*, rho guanine nucleotide exchange factor 12 gene; ASD, atrial septal defect; *CTNNB1*, catenin beta 1 gene; ECA, extracardiac anomaly; *KDR*, kinase insert domain receptor gene; LVOTO, left ventricular outflow tract obstruction; *MLLT10*, histone lysine methyltransferase DOT1L cofactor gene; NDD, neurodevelopmental delay; pLI, probability of loss-of-function intolerance; *PTEN*, phosphatase and tensin homolog gene; *TCF12*, transcription factor 12 gene; *TFRC*, transferrin receptor gene; ToF, tetralogy of Fallot; VATER, vertebral, anal, tracheal, esophageal, and renal abnormalities; VSD, ventricular septal defect.

## Discussion

In a large CHD cohort, we demonstrated an increased prevalence of LoF variants in CR genes, particularly those with established roles in CHD or that regulate gene expression (Table 1). Patients with CHD and variants in the CR genes were more likely to have ECA and/or NDD (eTable 9 in the Supplement), thereby prioritizing patients for prospective studies to determine if clinical outcomes validate CR. Importantly, we identified no increase rates of LoF in CR genes among patients with isolated CHD. Additionally, our data (Table 2) indicate that LoF variants in some CR genes contribute to CHD, prioritizing new molecules for mechanistic studies in heart development.

Recent genetic analyses^6,7,11,12^ demonstrate that many CHD genes function in regulating the epigenome and gene transcription and translation—processes that are critical to orchestrating cardiac progenitor cell proliferation, lineage commitment, and differentiation. These genes broadly participate in human development and harbor the highest rates of damaging variants among patients with CHD and ECA or NDD.^6,11^ Stem cells, with germline and somatic variants in genes with similar key functions, cause cancer.^13,14,15^ That subsets of patients with CHD (with ECA or NDD; eTable 9 in the Supplement) had the highest burden of LoF variants in these CR genes emphasizes the broad expression patterns and shared molecular mechanisms for some developmental defects and cancer. By contrast, damaging variants in genes with highly enriched expression in the heart, a tissue with exceedingly rare cancers, may preferentially cause isolated CHD and convey low CR.

Patients with CHD have common CR factors found in the general population, while our data indicate that CHD genotypes can contribute additional CR. Prior studies defined this association in a few CHD genes, including the lysine methyltransferase 2D (*MLL2/KMT2D*) gene, which encodes a chromatin modifier with broad tissue expression. Dominant de novo LoF variants in *MLL2/KMT2D* cause Kabuki syndrome with CHD,^12^ while somatic variants cause lung and colon adenocarcinomas.^8^ Our data extend these recognized linkages by defining more CR genes in more patients with CHD and indicating potentially shared disease mechanisms. For example, dysregulated vascular endothelial growth factor signaling from damaging variants in the kinase insert domain receptor (*KDR*) gene, which encodes a vascular endothelial growth factor receptor, could account for malformations of the great vessels^6,7^ and cancer-associated angiogenesis.

Biallelic variants are often required for oncogenesis,^8,14^ whereas 1 damaging variant is often sufficient to perturb cardiac morphogenesis. This raises the possibility that damaging variants in patients with CHD set the stage but do not directly initiate oncogenesis. Increased CR likely reflects additional, amplifying factors, including radiation and other CHD-associated exposures. Consistent with this model, we note that dominant *MLL2/KMT2D* variants cause CHD,^6,7,11^ while biallelic loss occurs in many cancers. We propose that CR is increased because the germline variant provides the first of 2 hits needed for cancer to emerge. Across this CHD cohort, 3.6% had LoF variants in recessive CR genes, while 1.8% had LoF variants in dominant CR genes. We speculate that high lifetime radiation doses might increase somatic variants that complement CHD LoF variants.

### Limitations

This study has limitations, including the young ages of the patients with CHD and the need for longitudinal cancer surveillance to benchmark the clinical relevance of CR variants in patients with CHD and help determine causal associations, as well as whether associations are modified by nongenetic factors. Control participants were considerably older, potentially introducing survivor bias that would enhance the burden of CR variants in patients with CHD. Depth of sequencing precluded differentiation of variants as germline or high-level mosaic. We analyzed only LoF variants since defining missense variants as damaging requires detailed functional assessments, and thus our data provide only a conservative estimate of CR variants in patients with CHD. This also precluded assessment of burden for CR genes that operate by gain-of-function mechanisms in cancer, such as protein tyrosine phosphatase non-receptor type 11 (*PTPN11*) and other RASopathy genes.^14^

## Conclusions

Decades of therapeutic progress enable long-term survival for newborns with CHD, and current estimates indicate 6 in 1000 adults are survivors of CHD.^1^ The recognition that CHD genotypes influence CR can promote clinical surveillance and early interventions and further promote lifelong health in adult patients with CHD. Additionally, we suggest that mechanistic studies into the molecular and cellular processes that are disrupted by damaging variants in CHD and cancer genes may uncover insights that inform new treatment strategies for both disorders.

## Notes

Supplement. **eMethods.** **eTable 1.** Cohort Characteristics **eTable 2.** Characteristics of all COSMIC Cancer Risk Genes **eTable 3.** Dominant CHD Genes that are also Cancer Risk Genes **eTable 4.** Loss of Function Variants in CHD Discovery and Replication Cohorts **eTable 5.** Discovery Cohort Analysis **eTable 6.** Replication Cohort Analysis **eTable 7.** CHD Phenotypes and CR Variant Status **eTable 8.** Per Gene Count of all Loss of Function Variants **eTable 9.** Cohort Subset Analysis **eTable 10.** Characteristics of genes enriched in CHD patients with congenital anomalies and/or neurodevelopmental delay **eReferences.**
