# Supplementary material for: Association of Damaging Variants in Genes With Increased Cancer Risk Among Patients With Congenital Heart Disease
Source: JAMA Cardiol. 2020 Oct 21;6(4):1–6. doi: 10.1001/jamacardio.2020.4947 (PMC7578917; doi:10.1001/jamacardio.2020.4947)
Supplement: Supplement. — eMethods. eTable 1. Cohort Characteristics eTable 2. Characteristics of all COSMIC Cancer Risk Genes eTable 3. Dominant CHD Genes that are also Cancer Risk Genes eTable 4. Loss of Function Variants in CHD Discovery and Replication Cohorts eTable 5. Discovery Cohort Analysis eTable 6. Replication Cohort Analysis eTable 7. CHD Phenotypes and CR Variant Status eTable 8. Per Gene Count of all Loss of Function Variants eTable 9. Cohort Subset Analysis eTable 10. Characteristics of genes enriched in CHD patients with congenital anomalies and/or neurodevelopmental delay eReferences. [file jamacardiol-e204947-s001.pdf]

Supplemental Online Content

Morton SU, Shimamura A, Newburger PE, et al. Association of damaging variants in genes with increased cancer risk among patients with congenital heart disease. *JAMA Cardiol.* Published online October 21, 2020. doi:10.1001/jamacardio.2020.4947

eMethods.
eTable 1. Cohort Characteristics
eTable 2. Characteristics of all COSMIC Cancer Risk Genes
eTable 3. Dominant CHD Genes that are also Cancer Risk Genes
eTable 4. Loss of Function Variants in CHD Discovery and Replication Cohorts
eTable 5. Discovery Cohort Analysis
eTable 6. Replication Cohort Analysis
eTable 7. CHD Phenotypes and CR Variant Status
eTable 8. Per Gene Count of all Loss of Function Variants
eTable 9. Cohort Subset Analysis
eTable 10. Characteristics of genes enriched in CHD patients with congenital anomalies and/or neurodevelopmental delay
eReferences.

This supplemental material has been provided by the authors to give readers additional information about their work.

© 2020 Morton SU et al. JAMA Cardiology.

eMethods
Participants

CHD Participants: CHD participants were recruited to the Congenital Heart Disease Network Study of the Pediatric Cardiac Genomics Consortium (CHD GENES: ClinicalTrials.gov identifier NCT01196182) or the Boston Children's Hospital Adult Congenital Heart Disease Biobank as previously described.1,2 All participants or their parents provided written informed consent using protocols that were reviewed and approved by institutional review boards at participating institutions. For this study, only CHD probands were included, exclusive of those with a clinically identified chromosomal aneuploidy or copy number variant that causes CHD. In total we excluded 64 PCGC participants with Trisomy 21 or 22q11 deletion syndrome; none had a LoF variant in a CR gene. Phenotype comparison between cohort subsets was performed using binomial p-values for 12 common CHD subtypes.

Control Participants: Controls for discovery and replication analyses came from two cohorts. The control cohort for discovery analyses are 3,578 parents (of at least two children, one with and one without autism),3 recruited into the Simons Simplex Collection (SSC)4 with written infomred consent. WES data on these parents was obtained from the SFARIbase with approval.

The control cohort for replication analyses included 6230 adults without psychiatric disorders who participated in the Sweden-Schizophrenia Population-Based Study5 and provided written informed consent. WES data (BWA-ALN/GATK v4.1 Variant Call File) was obtained from dbGaP with DAC approval.

Demographic features of cohorts are listed in eTable 1.

Identification of European participants

Participants with predominantly European ancestry were defined by principal component analysis (PCA) of single nucleotide polymorphisms (minor allele frequency > 0.05) from each cohort, merged with WES data from the 1000 Genomes Project Phase 3 release using Plink 1.9. Standard quality control measures for sample variant missingness, genotype variant missingness, and deviation from Hardy-Weinburg Equilibrium were applied. PCA was performed on ~96,000 shared common variants using Plink 1.9. Samples from CHD and controls cohorts that colocalized with non-Finnish European samples from the 1000 Genomes dataset were selected.

Cancer risk genes

723 cancer risk genes were identified from the Catalogue of Somatic Mutations in Cancer - Cancer Gene Consensus database (denoted COSMIC; eTable 2).6,7 Among these risk genes, 38 (5%) are known to be associated with human CHD in the Online Mendelian Inheritance in Man database8 (eTable 3), and 307 (42%) are highly expressed in the developing heart, defined by those genes in the top quartile of mRNA expression of the E14.5 mouse heart.9 The overlap between these gene lists was not more than expected by hypergeometric distribution (p-values 1 for both overlaps). The subset of 216 regulatory cancer risk genes that also participate in RNA transcription or mRNA processing were identified using the following GO annotations: DNA- binding transcription factor activity, mRNA processing, and transcription factor binding.

Variant filtering

We filtered rare LoF variants as previously reported using an analytic strategy optimized for detection of germline variants.10 In brief, candidate variants were filtered for rarity (allele frequency ≤ 10-5 across all WES samples in gnomAD) and high-quality heterozygotes (pass GATK VQSR with truth sensitivity threshold of 99.5 for both SNPs and indels, minimum eight total reads, GQ score ≥ 20 per variant, mapping quality [MQ] score ≥ 59, PS < 60, GD>2, and

© 2020 Morton SU et al. JAMA Cardiology.

minimum 20% alternate allele ratio in the proband if alternate allele reads ≥ 10 or, if alternate allele reads is < 10, a minimum 28% alternate ratio). All candidate *de novo* variants (DNVs) were confirmed by IGV visualization. LoF variants were defined as any variants causing premature stop codons (frameshift or stop-gain), start loss, or altering canonical splice sites, as annotated by SNPEFF.11 Additionally, variants located in segmental duplication regions (as annotated by ANNOVAR12) were excluded. CHD probands were identified as having an OMIM CHD gene variant if they possessed a variant within one of the 114 dominant OMIM CHD genes (eTable 2). Of the eight LoF variants were identified in CR genes on chromosome X, seven occurred in female participants.

Datasets containing all LoF variants in for CHD patients (discovery and replication cohorts) are provided on eTable 4. Comparable datasets for non-CHD control subjects are available on request.

Gene-set enrichment analysis
A one-sided binomial test was conducted by comparing the proportion of CHD participants *versus* non-CHD participants with rare LoF variants among sets genes of interest. Threshold p-values were determined using a Bonferroni correction for the number of independent tests per table. This comparison was repeated for subsets of the CHD cohort (eTable 9), applying Bonferroni correction for the number of tests. One-sided binomial comparison was also performed for all genes without Bonferroni correction (eTable 8). Candidate genes were identified as having genetic constraint (gnomAD pLI > 0.05)13, being in the upper quartile of genes expressed in the E14.5 mouse heart,9 a one-sided binomial p-value <0.05 for CHD vs non-CHD LoF variants, and at least 2 CHD LoF variants (eTable 11).

Gene ontology analysis
Enrichment for biological pathway functions among the 119 CR genes with LoF variants in patients with isolated CHD and among the 238 genes with LoF variants in CHD ± ECA or NDD was queried using PANTHER14 (pantherdb.org). We compared the frequency of gene ontology terms for between CR genes in isolated CHD and CR genes in CHD ± ECA or NDD. Significant enrichments were determined as those with a False Discovery Rate p <0.05.

© 2020 Morton SU et al. JAMA Cardiology.

eTables

eTable 1. Cohort Characteristics

	CHD		Control	
	Discovery	Replication	Discovery	Replication
**N**	2222	2221	3578	6230
**Male, N (%)**	1126 (59)	1099 (58)	1789 (50)	6543 (52)
**Mean Age in Years (Standard Deviation, Range)**	12.8 (14.7, 0-84)	13.2 (14.9, 0-78)	44.8 (6.1, 27.2-68.1)	56.3 (11.5, 21.0-92.0)
**CHD Type, N (%)**				
Conotruncal defects (including tetralogy of Fallot, transposition of the great arteries, truncus arteriosus)	655 (29)	654 (29)	-	-
Left outflow tract obstruction	519 (23)	501 (23)	-	-
Right outflow tract obstruction	49 (2)	73 (3)	-	-
Endocardial cushion defect	85 (4)	97 (4)		
Other	914 (41)	896 (40)	-	-
**Neurodevelopmental Delay, N (%)**				
Yes	713 (32)	680(31)	-	-
No	452 (20)	498 (22)	-	-
Unknown	1057 (48)	1043 (47)	-	-
**Extracardiac Anomalies, N (%)**				
Yes	739 (33)	743 (33)	-	-
No	1178 (53)	1162 (52)	-	-
Unknown	305 (14)	316 (14)	-	-

© 2020 Morton SU et al. JAMA Cardiology.

minimum 20% alternate allele ratio in the proband if alternate allele reads ≥ 10 or, if alternate allele reads is < 10, a minimum 28% alternate ratio). All candidate *de novo* variants (DNVs) were confirmed by IGV visualization. LoF variants were defined as any variants causing premature stop codons (frameshift or stop-gain), start loss, or altering canonical splice sites, as annotated by SNPEFF.11 Additionally, variants located in segmental duplication regions (as annotated by ANNOVAR12) were excluded. CHD probands were identified as having an OMIM CHD gene variant if they possessed a variant within one of the 114 dominant OMIM CHD genes (eTable 2). Of the eight LoF variants were identified in CR genes on chromosome X, seven occurred in female participants.

Datasets containing all LoF variants in for CHD patients (discovery and replication cohorts) are provided on eTable 4. Comparable datasets for non-CHD control subjects are available on request.

Gene-set enrichment analysis

A one-sided binomial test was conducted by comparing the proportion of CHD participants *versus* non-CHD participants with rare LoF variants among sets genes of interest. Threshold p-values were determined using a Bonferroni correction for the number of independent tests per table. This comparison was repeated for subsets of the CHD cohort (eTable 9), applying Bonferroni correction for the number of tests. One-sided binomial comparison was also performed for all genes without Bonferroni correction (eTable 8). Candidate genes were identified as having genetic constraint (gnomAD pLI > 0.05)13, being in the upper quartile of genes expressed in the E14.5 mouse heart,9 a one-sided binomial p-value <0.05 for CHD vs non-CHD LoF variants, and at least 2 CHD LoF variants (eTable 11).

Gene ontology analysis

Enrichment for biological pathway functions among the 119 CR genes with LoF variants in patients with isolated CHD and among the 238 genes with LoF variants in CHD ± ECA or NDD was queried using PANTHER14 (pantherdb.org). We compared the frequency of gene ontology terms for between CR genes in isolated CHD and CR genes in CHD ± ECA or NDD. Significant enrichments were determined as those with a False Discovery Rate p <0.05.

© 2020 Morton SU et al. JAMA Cardiology.

eTables

eTable 1. Cohort Characteristics

	**CHD**		**Control**	
	**Discovery**	**Replication**	**Discovery**	**Replication**
**N**	2222	2221	3578	6230
**Male, N (%)**	1126 (59)	1099 (58)	1789 (50)	6543 (52)
**Mean Age in Years (Standard Deviation, Range)**	12.8 (14.7, 0-84)	13.2 (14.9, 0-78)	44.8 (6.1, 27.2-68.1)	56.3 (11.5, 21.0-92.0)
**CHD Type, N (%)**				
Conotruncal defects (including tetralogy of Fallot, transposition of the great arteries, truncus arteriosus)	655 (29)	654 (29)	-	-
Left outflow tract obstruction	519 (23)	501 (23)	-	-
Right outflow tract obstruction	49 (2)	73 (3)	-	-
Endocardial cushion defect	85 (4)	97 (4)		
Other	914 (41)	896 (40)	-	-
**Neurodevelopmental Delay, N (%)**				
Yes	713 (32)	680(31)	-	-
No	452 (20)	498 (22)	-	-
Unknown	1057 (48)	1043 (47)	-	-
**Extracardiac Anomalies, N (%)**				
Yes	739 (33)	743 (33)	-	-
No	1178 (53)	1162 (52)	-	-
Unknown	305 (14)	316 (14)	-	-

© 2020 Morton SU et al. JAMA Cardiology.

eTable 2. Characteristics of all COSMIC cancer risk genes

Gene Symbol	Somatic	Germline	Molecular Genetics	OMIM CHD Gene	Regulatory Function
A1CF	yes			FALSE	TRUE
ABI1	yes		Dominant	FALSE	FALSE
ABL1	yes		Dominant	FALSE	FALSE
ABL2	yes		Dominant	FALSE	FALSE
ACKR3	yes		Dominant	FALSE	FALSE
ACSL3	yes		Dominant	FALSE	FALSE
ACSL6	yes		Dominant	FALSE	FALSE
ACVR1	yes		Dominant	FALSE	FALSE
ACVR2A	yes		Recurrent	FALSE	FALSE
AFF1	yes		Dominant	FALSE	TRUE
AFF3	yes		Dominant	FALSE	TRUE
AFF4	yes		Dominant	FALSE	TRUE
AKAP9	yes		Dominant	FALSE	FALSE
AKT1	yes		Dominant	FALSE	FALSE
AKT2	yes		Dominant	FALSE	FALSE
AKT3	yes			FALSE	FALSE
ALDH2	yes		Dominant	FALSE	FALSE
ALK	yes	yes	Dominant	FALSE	FALSE
AMER1	yes		Recurrent	FALSE	FALSE
ANK1	yes			FALSE	FALSE
APC	yes	yes	Recurrent	FALSE	FALSE
APOBEC3B		yes	Recurrent	FALSE	FALSE
AR	yes	yes	Dominant	FALSE	TRUE
ARAF	yes			FALSE	FALSE
ARHGAP26	yes		Dominant	FALSE	FALSE
ARHGAP5	yes			FALSE	FALSE
ARHGEF10	yes			FALSE	FALSE
ARHGEF10L	yes			FALSE	FALSE
ARHGEF12	yes		Dominant	FALSE	FALSE
ARID1A	yes		Recurrent	TRUE	TRUE
ARID1B	yes		Recurrent	FALSE	FALSE
ARID2	yes		Recurrent	FALSE	FALSE
ARNT	yes		Dominant	FALSE	TRUE
ASPSCR1	yes		Dominant	FALSE	FALSE
ASXL1	yes		Recurrent	TRUE	TRUE
ASXL2	yes			FALSE	FALSE
ATF1	yes		Dominant	FALSE	TRUE
ATIC	yes		Dominant	TRUE	FALSE
ATM	yes	yes	Recurrent	FALSE	FALSE
ATP1A1	yes		Dominant	FALSE	FALSE
ATP2B3	yes		Dominant	FALSE	FALSE
ATR	yes	yes	Dominant	FALSE	FALSE
ATRX	yes		Recurrent	FALSE	FALSE
AXIN1	yes		Recurrent	FALSE	FALSE
AXIN2	yes	yes	Recurrent	FALSE	FALSE
B2M	yes		Recurrent	FALSE	FALSE
BAP1	yes	yes	Recurrent	FALSE	FALSE
BARD1	yes	yes		FALSE	FALSE
BAX	yes			FALSE	FALSE
BAZ1A	yes			FALSE	FALSE
BCL10	yes		Dominant	FALSE	TRUE
BCL11A	yes		Dominant	FALSE	TRUE

© 2020 Morton SU et al. JAMA Cardiology.

Gene Symbol	Somatic	Germline	Molecular Genetics	OMIM CHD Gene	Regulatory Function
BCL11B	yes		Dominant	FALSE	TRUE
BCL2	yes		Dominant	FALSE	FALSE
BCL2L12	yes			FALSE	FALSE
BCL3	yes		Dominant	FALSE	TRUE
BCL6	yes		Dominant	FALSE	TRUE
BCL7A	yes		Dominant	FALSE	FALSE
BCL9	yes		Dominant	FALSE	FALSE
BCL9L	yes			FALSE	FALSE
BCLAF1	yes			FALSE	FALSE
BCOR	yes		Recurrent	TRUE	FALSE
BCORL1	yes			FALSE	FALSE
BCR	yes		Dominant	FALSE	FALSE
BIRC3	yes		Dominant	FALSE	FALSE
BIRC6	yes			FALSE	FALSE
BLM		yes	Recurrent	FALSE	FALSE
BMP5	yes			FALSE	FALSE
BMPR1A		yes	Recurrent	FALSE	TRUE
BRAF	yes		Dominant	FALSE	FALSE
BRCA1	yes	yes	Recurrent	FALSE	TRUE
BRCA2	yes	yes	Recurrent	FALSE	FALSE
BRD3	yes		Dominant	FALSE	FALSE
BRD4	yes		Dominant	FALSE	TRUE
BRIP1		yes	Recurrent	FALSE	FALSE
BTG1	yes		Dominant	FALSE	FALSE
BTK	yes		Dominant	FALSE	FALSE
BUB1B		yes	Recurrent	FALSE	FALSE
C15orf65	yes		Dominant	FALSE	FALSE
C2orf44	yes		Dominant	FALSE	FALSE
CACNA1D	yes		Dominant	FALSE	FALSE
CALR	yes		Dominant	FALSE	TRUE
CAMTA1	yes		Dominant	FALSE	TRUE
CANT1	yes		Dominant	FALSE	FALSE
CARD11	yes		Dominant	FALSE	FALSE
CARS	yes		Dominant	FALSE	FALSE
CASC5	yes		Dominant	FALSE	FALSE
CASP3	yes			FALSE	FALSE
CASP8	yes		Recurrent	FALSE	FALSE
CASP9	yes			FALSE	FALSE
CBFA2T3	yes		Dominant	FALSE	TRUE
CBFB	yes		Dominant	FALSE	TRUE
CBL	yes		Dominant/Recurrent	TRUE	FALSE
CBLB	yes		Recurrent	FALSE	FALSE
CBLC	yes		Recurrent	FALSE	FALSE
CCDC6	yes		Dominant	FALSE	FALSE
CCNB1IP1	yes		Dominant	FALSE	FALSE
CCNC	yes			FALSE	FALSE
CCND1	yes		Dominant	FALSE	TRUE
CCND2	yes		Dominant	FALSE	FALSE
CCND3	yes		Dominant	FALSE	FALSE
CCNE1	yes		Dominant	FALSE	TRUE
CCR4	yes			FALSE	FALSE
CCR7	yes			FALSE	FALSE

© 2020 Morton SU et al. JAMA Cardiology.

Gene Symbol	Somatic	Germline	Molecular Genetics	OMIM CHD Gene	Regulatory Function
CD209	yes			FALSE	FALSE
CD274	yes		Dominant	FALSE	FALSE
CD28	yes			FALSE	FALSE
CD74	yes		Dominant	FALSE	FALSE
CD79A	yes		Dominant	FALSE	FALSE
CD79B	yes		Dominant	FALSE	FALSE
CDC73	yes	yes	Recurrent	FALSE	TRUE
CDH1	yes	yes	Recurrent	FALSE	FALSE
CDH10	yes	yes		FALSE	FALSE
CDH11	yes		Dominant	FALSE	FALSE
CDH17	yes			FALSE	FALSE
CDK12	yes		Recurrent	FALSE	TRUE
CDK4		yes	Dominant	FALSE	FALSE
CDK6	yes		Dominant	FALSE	FALSE
CDKN1A	yes			FALSE	FALSE
CDKN1B	yes	yes	Recurrent	FALSE	FALSE
CDKN2A	yes	yes	Recurrent	FALSE	TRUE
CDKN2C	yes		Recurrent	FALSE	FALSE
CDX2	yes		Dominant	FALSE	TRUE
CEBPA	yes		Dominant	FALSE	TRUE
CEP89	yes		Dominant	FALSE	FALSE
CHCHD7	yes		Dominant	FALSE	FALSE
CHD2	yes			FALSE	FALSE
CHD4	yes		Dominant	FALSE	TRUE
CHEK2		yes	Recurrent	FALSE	FALSE
CHIC2	yes		Dominant	FALSE	FALSE
CHST11	yes			FALSE	FALSE
CIC	yes		Recurrent	FALSE	TRUE
CIITA	yes		Dominant	FALSE	TRUE
CLIP1	yes		Dominant	FALSE	FALSE
CLP1	yes		Dominant	FALSE	TRUE
CLTC	yes		Dominant	FALSE	FALSE
CLTCL1	yes		Dominant	FALSE	FALSE
CNBD1	yes			FALSE	FALSE
CNBP	yes		Dominant	FALSE	FALSE
CNOT3	yes		Recurrent	FALSE	FALSE
CNTNAP2	yes			FALSE	FALSE
CNTRL	yes		Dominant	FALSE	FALSE
COL1A1	yes		Dominant	TRUE	FALSE
COL2A1	yes		Recurrent	TRUE	FALSE
COL3A1	yes			TRUE	FALSE
COX6C	yes		Dominant	FALSE	FALSE
CPEB3	yes			FALSE	TRUE
CREB1	yes		Dominant	FALSE	TRUE
CREB3L1	yes		Dominant	FALSE	TRUE
CREB3L2	yes		Dominant	FALSE	TRUE
CREBBP	yes		Dominant/Recurrent	TRUE	TRUE
CRLF2	yes		Dominant	FALSE	FALSE
CRNKL1	yes			FALSE	TRUE
CRTC1	yes		Dominant	FALSE	TRUE
CRTC3	yes		Dominant	FALSE	TRUE
CSF1R	yes			FALSE	FALSE

© 2020 Morton SU et al. JAMA Cardiology.

Gene Symbol	Somatic	Germline	Molecular Genetics	OMIM CHD Gene	Regulatory Function
CSF3R	yes		Dominant	FALSE	FALSE
CSMD3	yes			FALSE	FALSE
CTCF	yes		Dominant	FALSE	TRUE
CTNNA2	yes			FALSE	FALSE
CTNNB1	yes		Dominant	FALSE	TRUE
CTNND1	yes			FALSE	FALSE
CTNND2	yes			FALSE	FALSE
CUL3	yes			FALSE	FALSE
CUX1	yes		Dominant	FALSE	FALSE
CXCR4	yes	yes	Dominant	FALSE	FALSE
CYLD	yes	yes	Recurrent	FALSE	FALSE
CYP2C8	yes			FALSE	FALSE
CYSLTR2	yes			FALSE	FALSE
DAXX	yes		Recurrent	FALSE	TRUE
DCAF12L2	yes			FALSE	FALSE
DCC	yes			FALSE	FALSE
DCTN1	yes		Dominant	FALSE	FALSE
DDB2		yes	Recurrent	FALSE	FALSE
DDIT3	yes		Dominant	FALSE	TRUE
DDR2	yes		Dominant	FALSE	FALSE
DDX10	yes		Dominant	FALSE	FALSE
DDX3X	yes			FALSE	TRUE
DDX5	yes		Dominant	FALSE	TRUE
DDX6	yes		Dominant	FALSE	FALSE
DEK	yes		Dominant	FALSE	FALSE
DGCR8	yes			FALSE	FALSE
DICER1	yes	yes	Recurrent	FALSE	FALSE
DNAJB1	yes		Dominant	FALSE	FALSE
DNM2	yes		Recurrent	FALSE	FALSE
DNMT3A	yes		Dominant	FALSE	FALSE
DROSHA	yes			FALSE	FALSE
DUX4L1	yes		Dominant	FALSE	FALSE
EBF1	yes		Dominant	FALSE	TRUE
ECT2L	yes		Recurrent	FALSE	FALSE
EED	yes			FALSE	FALSE
EGFR	yes	yes	Dominant	FALSE	FALSE
EIF1AX	yes			FALSE	FALSE
EIF3E	yes		Dominant	FALSE	FALSE
EIF4A2	yes		Dominant	FALSE	FALSE
ELF3	yes			FALSE	TRUE
ELF4	yes		Dominant	FALSE	TRUE
ELK4	yes		Dominant	FALSE	TRUE
ELL	yes		Dominant	FALSE	FALSE
ELN	yes		Dominant	TRUE	FALSE
EML4	yes		Dominant	FALSE	FALSE
EP300	yes		Recurrent	FALSE	TRUE
EPAS1	yes		Dominant	FALSE	TRUE
EPHA3	yes			FALSE	FALSE
EPHA7	yes			FALSE	FALSE
EPS15	yes		Dominant	FALSE	FALSE
ERBB2	yes		Dominant	FALSE	FALSE
ERBB3	yes		Dominant	FALSE	FALSE

© 2020 Morton SU et al. JAMA Cardiology.

Gene Symbol	Somatic	Germline	Molecular Genetics	OMIM CHD Gene	Regulatory Function
ERBB4	yes	yes	Dominant	FALSE	FALSE
ERC1	yes		Dominant	FALSE	FALSE
ERCC2		yes	Recurrent	FALSE	TRUE
ERCC3		yes	Recurrent	FALSE	TRUE
ERCC4		yes	Recurrent	FALSE	TRUE
ERCC5		yes	Recurrent	FALSE	FALSE
ERG	yes		Dominant	FALSE	TRUE
ESR1	yes		Dominant	FALSE	TRUE
ETNK1	yes		Dominant	FALSE	FALSE
ETV1	yes		Dominant	FALSE	TRUE
ETV4	yes		Dominant	FALSE	TRUE
ETV5	yes		Dominant	FALSE	TRUE
ETV6	yes		Dominant	FALSE	FALSE
EWSR1	yes		Dominant	FALSE	FALSE
EXT1		yes	Recurrent	FALSE	FALSE
EXT2		yes	Recurrent	FALSE	FALSE
EZH2	yes		Dominant	FALSE	TRUE
EZR	yes		Dominant	FALSE	FALSE
FAM131B	yes		Dominant	FALSE	FALSE
FAM135B	yes			FALSE	FALSE
FAM46C	yes		Recurrent	FALSE	FALSE
FAM47C	yes			FALSE	FALSE
FANCA		yes	Recurrent	FALSE	FALSE
FANCC		yes	Recurrent	FALSE	FALSE
FANCD2		yes	Recurrent	FALSE	FALSE
FANCE		yes	Recurrent	FALSE	FALSE
FANCF		yes	Recurrent	FALSE	FALSE
FANCG		yes	Recurrent	FALSE	FALSE
FAS	yes		Recurrent	FALSE	FALSE
FAT1	yes	yes		FALSE	FALSE
FAT3	yes			FALSE	FALSE
FAT4	yes			FALSE	FALSE
FBLN2	yes			FALSE	FALSE
FBXO11	yes		Recurrent	FALSE	FALSE
FBXW7	yes		Recurrent	FALSE	FALSE
FCGR2B	yes		Dominant	FALSE	FALSE
FCRL4	yes		Dominant	FALSE	FALSE
FEN1		yes		FALSE	FALSE
FES	yes			FALSE	FALSE
FEV	yes		Dominant	FALSE	FALSE
FGFR1	yes		Dominant	TRUE	FALSE
FGFR1OP	yes		Dominant	FALSE	FALSE
FGFR2	yes		Dominant	FALSE	FALSE
FGFR3	yes		Dominant	FALSE	FALSE
FGFR4	yes		Dominant	FALSE	FALSE
FH		yes	Recurrent	FALSE	FALSE
FHIT	yes		Dominant	FALSE	FALSE
FIP1L1	yes		Dominant	FALSE	TRUE
FKBP9	yes			FALSE	FALSE
FLCN		yes	Recurrent	FALSE	FALSE
FLI1	yes		Dominant	FALSE	TRUE
FLNA	yes			TRUE	TRUE

© 2020 Morton SU et al. JAMA Cardiology.

Gene Symbol	Somatic	Germline	Molecular Genetics	OMIM CHD Gene	Regulatory Function
FLT3	yes		Dominant	FALSE	TRUE
FLT4	yes		Dominant	FALSE	FALSE
FNBP1	yes		Dominant	FALSE	FALSE
FOXA1	yes		Dominant	FALSE	TRUE
FOXL2	yes		Dominant	FALSE	TRUE
FOXO1	yes		Dominant	FALSE	TRUE
FOXO3	yes		Dominant	FALSE	TRUE
FOXO4	yes		Dominant	FALSE	TRUE
FOXP1	yes		Dominant	FALSE	FALSE
FOXR1	yes			FALSE	TRUE
FSTL3	yes		Dominant	FALSE	FALSE
FUBP1	yes		Recurrent	FALSE	TRUE
FUS	yes		Dominant	FALSE	TRUE
GAS7	yes		Dominant	FALSE	FALSE
GATA1	yes		Dominant	FALSE	TRUE
GATA2	yes		Dominant	FALSE	FALSE
GATA3	yes		Recurrent	FALSE	TRUE
GLI1	yes			FALSE	TRUE
GMPS	yes		Dominant	FALSE	FALSE
GNA11	yes		Dominant	FALSE	FALSE
GNAQ	yes		Dominant	FALSE	FALSE
GNAS	yes		Dominant	FALSE	FALSE
GOLGA5	yes		Dominant	FALSE	FALSE
GOPC	yes		Dominant	FALSE	FALSE
GPC3		yes	Recurrent/X	TRUE	FALSE
GPC5	yes			FALSE	FALSE
GPHN	yes		Dominant	FALSE	FALSE
GRIN2A	yes		Recurrent	FALSE	FALSE
GRM3	yes			FALSE	FALSE
H3F3A	yes		Dominant	FALSE	FALSE
H3F3B	yes		Dominant	FALSE	FALSE
HERPUD1	yes		Dominant	FALSE	FALSE
HEY1	yes		Dominant	FALSE	TRUE
HIF1A	yes		Dominant	FALSE	TRUE
HIP1	yes		Dominant	FALSE	FALSE
HIST1H3B	yes		Dominant	FALSE	FALSE
HIST1H4I	yes		Dominant	FALSE	FALSE
HLA-A	yes		Dominant	FALSE	FALSE
HLF	yes		Dominant	FALSE	TRUE
HMGA1	yes		Dominant	FALSE	TRUE
HMGA2	yes		Dominant	FALSE	TRUE
HMGN2P46	yes		Dominant	FALSE	FALSE
HNF1A	yes	yes	Recurrent	FALSE	TRUE
HNRNPA2B1	yes		Dominant	FALSE	TRUE
HOOK3	yes		Dominant	FALSE	FALSE
HOXA11	yes		Dominant	FALSE	TRUE
HOXA13	yes		Dominant	FALSE	TRUE
HOXA9	yes		Dominant	FALSE	TRUE
HOXC11	yes		Dominant	FALSE	TRUE
HOXC13	yes		Dominant	FALSE	TRUE
HOXD11	yes		Dominant	FALSE	TRUE
HOXD13	yes		Dominant	FALSE	TRUE

© 2020 Morton SU et al. JAMA Cardiology.

Gene Symbol	Somatic	Germline	Molecular Genetics	OMIM CHD Gene	Regulatory Function
HRAS	yes	yes	Dominant	TRUE	FALSE
HSP90AA1	yes		Dominant	FALSE	FALSE
HSP90AB1	yes		Dominant	FALSE	FALSE
ID3	yes			FALSE	TRUE
IDH1	yes		Dominant	FALSE	FALSE
IDH2	yes		Dominant	FALSE	FALSE
IGF2BP2	yes			FALSE	FALSE
IGH	yes		Dominant	FALSE	FALSE
IGK	yes		Dominant	FALSE	FALSE
IGL	yes		Dominant	FALSE	FALSE
IKBKB	yes		Dominant	FALSE	FALSE
IKZF1	yes		Dominant/Recurrent	FALSE	TRUE
IL2	yes		Dominant	FALSE	FALSE
IL21R	yes		Dominant	FALSE	FALSE
IL6ST	yes		Dominant	FALSE	FALSE
IL7R	yes		Dominant	FALSE	FALSE
IRF4	yes		Dominant	FALSE	TRUE
IRS4	yes			FALSE	FALSE
ISX	yes			FALSE	TRUE
ITGAV	yes			FALSE	FALSE
ITK	yes		Dominant	FALSE	FALSE
JAK1	yes		Dominant	FALSE	FALSE
JAK2	yes		Dominant	FALSE	FALSE
JAK3	yes		Dominant	FALSE	FALSE
JAZF1	yes		Dominant	FALSE	FALSE
JUN	yes		Dominant	FALSE	TRUE
KAT6A	yes		Dominant	FALSE	FALSE
KAT6B	yes		Dominant	TRUE	TRUE
KAT7	yes			FALSE	TRUE
KCNJ5	yes		Dominant	FALSE	FALSE
KDM5A	yes		Dominant	FALSE	FALSE
KDM5C	yes		Recurrent	FALSE	FALSE
KDM6A	yes		Recurrent	FALSE	FALSE
KDR	yes	yes	Dominant	FALSE	FALSE
KDSR	yes		Dominant	FALSE	FALSE
KEAP1	yes			FALSE	TRUE
KIAA1549	yes		Dominant	FALSE	FALSE
KIAA1598	yes		Dominant	FALSE	FALSE
KIF5B	yes		Dominant	FALSE	FALSE
KIT	yes	yes	Dominant	FALSE	FALSE
KLF4	yes		Dominant	FALSE	TRUE
KLF6	yes		Recurrent	FALSE	TRUE
KLK2	yes		Dominant	FALSE	FALSE
KMT2A	yes		Dominant	TRUE	TRUE
KMT2C	yes		Recurrent	FALSE	FALSE
KMT2D	yes		Recurrent	TRUE	TRUE
KNSTRN	yes			FALSE	FALSE
KRAS	yes		Dominant	TRUE	FALSE
KTN1	yes		Dominant	FALSE	FALSE
LARP4B	yes			FALSE	FALSE
LASP1	yes		Dominant	FALSE	FALSE
LATS1	yes			FALSE	TRUE

© 2020 Morton SU et al. JAMA Cardiology.

Gene Symbol	Somatic	Germline	Molecular Genetics	OMIM CHD Gene	Regulatory Function
LATS2	yes			FALSE	FALSE
LCK	yes		Dominant	FALSE	FALSE
LCP1	yes		Dominant	FALSE	FALSE
LEF1	yes			FALSE	TRUE
LEPROTL1	yes			FALSE	FALSE
LHFP	yes		Dominant	FALSE	FALSE
LIFR	yes		Dominant	FALSE	FALSE
LMNA	yes		Dominant	FALSE	FALSE
LMO1	yes	yes	Dominant	FALSE	FALSE
LMO2	yes		Dominant	FALSE	TRUE
LPP	yes		Dominant	FALSE	FALSE
LRIG3	yes		Dominant	FALSE	FALSE
LRP1B	yes		Recurrent	FALSE	FALSE
LSM14A	yes		Dominant	FALSE	FALSE
LYL1	yes		Dominant	FALSE	TRUE
LZTR1	yes	yes	Dominant	FALSE	TRUE
MACC1	yes			FALSE	TRUE
MAF	yes		Dominant	FALSE	TRUE
MAFB	yes		Dominant	FALSE	TRUE
MALAT1	yes		Dominant	FALSE	FALSE
MALT1	yes		Dominant	FALSE	FALSE
MAML2	yes		Dominant	FALSE	FALSE
MAP2K1	yes		Dominant	TRUE	FALSE
MAP2K2	yes		Dominant	TRUE	FALSE
MAP2K4	yes		Recurrent	FALSE	FALSE
MAP3K1	yes		Recurrent	FALSE	FALSE
MAP3K13	yes		Recurrent	FALSE	FALSE
MAPK1	yes		Dominant	FALSE	TRUE
MAX	yes	yes	Recurrent	FALSE	TRUE
MB21D2	yes			FALSE	FALSE
MDM2	yes		Dominant	FALSE	FALSE
MDM4	yes		Dominant	FALSE	FALSE
MDS2	yes		Dominant	FALSE	FALSE
MECOM	yes		Dominant	FALSE	TRUE
MED12	yes		Dominant	TRUE	TRUE
MEN1	yes	yes	Recurrent	FALSE	FALSE
MET	yes		Dominant	FALSE	FALSE
MGMT	yes			FALSE	FALSE
MITF	yes		Dominant	FALSE	TRUE
MKL1	yes		Dominant	FALSE	FALSE
MLF1	yes		Dominant	FALSE	FALSE
MLH1	yes	yes	Recurrent	FALSE	FALSE
MLLT1	yes		Dominant	FALSE	TRUE
MLLT10	yes		Dominant	FALSE	TRUE
MLLT11	yes		Dominant	FALSE	FALSE
MLLT3	yes		Dominant	FALSE	TRUE
MLLT4	yes		Dominant	FALSE	FALSE
MLLT6	yes		Dominant	FALSE	FALSE
MN1	yes		Dominant	FALSE	FALSE
MNX1	yes		Dominant	FALSE	TRUE
MPL	yes	yes	Dominant	FALSE	FALSE
MSH2	yes	yes	Recurrent	FALSE	FALSE

© 2020 Morton SU et al. JAMA Cardiology.

Gene Symbol	Somatic	Germline	Molecular Genetics	OMIM CHD Gene	Regulatory Function
MSH6	yes	yes	Recurrent	FALSE	FALSE
MSI2	yes		Dominant	FALSE	FALSE
MSN	yes		Dominant	FALSE	FALSE
MTCP1	yes		Dominant	FALSE	FALSE
MTOR	yes		Dominant	FALSE	TRUE
MUC1	yes		Dominant	FALSE	FALSE
MUC16	yes			FALSE	FALSE
MUC4	yes			FALSE	FALSE
MUTYH		yes	Recurrent	FALSE	FALSE
MYB	yes		Dominant	FALSE	TRUE
MYC	yes		Dominant	FALSE	TRUE
MYCL	yes		Dominant	FALSE	TRUE
MYCN	yes		Dominant	FALSE	TRUE
MYD88	yes		Dominant	FALSE	FALSE
MYH11	yes		Dominant	FALSE	FALSE
MYH9	yes		Dominant	FALSE	FALSE
MYO5A	yes		Dominant	FALSE	FALSE
MYOD1	yes		Dominant	FALSE	TRUE
N4BP2	yes			FALSE	FALSE
NAB2	yes		Dominant	FALSE	FALSE
NACA	yes		Dominant	FALSE	FALSE
NBEA	yes			FALSE	FALSE
NBN		yes	Recurrent	FALSE	TRUE
NCKIPSD	yes		Dominant	FALSE	FALSE
NCOA1	yes		Dominant	FALSE	TRUE
NCOA2	yes		Dominant	FALSE	TRUE
NCOA4	yes		Dominant	FALSE	TRUE
NCOR1	yes		Recurrent	FALSE	TRUE
NCOR2	yes		Dominant	FALSE	FALSE
NDRG1	yes		Dominant	FALSE	FALSE
NF1	yes	yes	Recurrent	TRUE	FALSE
NF2	yes	yes	Recurrent	FALSE	FALSE
NFATC2	yes		Dominant	FALSE	TRUE
NFE2L2	yes		Dominant	FALSE	TRUE
NFIB	yes		Dominant	FALSE	TRUE
NFKB2	yes		Dominant	FALSE	TRUE
NFKBIE	yes		Dominant/Recurrent	FALSE	FALSE
NIN	yes		Dominant	FALSE	FALSE
NKX2-1	yes		Dominant	FALSE	TRUE
NONO	yes		Dominant	FALSE	FALSE
NOTCH1	yes		Dominant/Recurrent	TRUE	TRUE
NOTCH2	yes		Dominant/Recurrent	TRUE	TRUE
NPM1	yes		Dominant	FALSE	TRUE
NR4A3	yes		Dominant	FALSE	TRUE
NRAS	yes		Dominant	TRUE	FALSE
NRG1	yes		Dominant	FALSE	FALSE
NSD1	yes		Dominant	TRUE	TRUE
NT5C2	yes		Dominant	FALSE	FALSE
NTHL1		yes		FALSE	FALSE
NTRK1	yes		Dominant	FALSE	FALSE
NTRK3	yes		Dominant	FALSE	FALSE
NUMA1	yes		Dominant	FALSE	FALSE

© 2020 Morton SU et al. JAMA Cardiology.

Gene Symbol	Somatic	Germline	Molecular Genetics	OMIM CHD Gene	Regulatory Function
NUP214	yes		Dominant	FALSE	FALSE
NUP98	yes		Dominant	FALSE	TRUE
NUTM1	yes		Dominant	FALSE	FALSE
NUTM2A	yes		Dominant	FALSE	FALSE
NUTM2B	yes		Dominant	FALSE	FALSE
OLIG2	yes		Dominant	FALSE	TRUE
OMD	yes		Dominant	FALSE	FALSE
P2RY8	yes		Dominant	FALSE	FALSE
PABPC1	yes			FALSE	TRUE
PAFAH1B2	yes		Dominant	FALSE	FALSE
PALB2		yes	Recurrent	FALSE	FALSE
PAX3	yes		Dominant	FALSE	TRUE
PAX5	yes		Dominant	FALSE	TRUE
PAX7	yes		Dominant	FALSE	TRUE
PAX8	yes		Dominant	FALSE	TRUE
PBRM1	yes		Recurrent	FALSE	FALSE
PBX1	yes		Dominant	FALSE	TRUE
PCBP1	yes			FALSE	TRUE
PCM1	yes		Dominant	FALSE	FALSE
PDCD1LG2	yes		Dominant	FALSE	FALSE
PDE4DIP	yes		Dominant	FALSE	FALSE
PDGFB	yes		Dominant	FALSE	FALSE
PDGFRA	yes	yes	Dominant	FALSE	FALSE
PDGFRB	yes		Dominant	FALSE	FALSE
PER1	yes		Dominant	FALSE	TRUE
PHF6	yes		Recurrent	FALSE	FALSE
PHOX2B	yes	yes	Recurrent	FALSE	TRUE
PICALM	yes		Dominant	FALSE	FALSE
PIK3CA	yes		Dominant	FALSE	FALSE
PIK3CB	yes			FALSE	FALSE
PIK3R1	yes		Recurrent	FALSE	TRUE
PIM1	yes		Dominant	FALSE	TRUE
PLAG1	yes		Dominant	FALSE	TRUE
PLCG1	yes		Dominant	FALSE	FALSE
PML	yes		Dominant	FALSE	FALSE
PMS1		yes	Recurrent	FALSE	FALSE
PMS2		yes	Recurrent	FALSE	FALSE
POLD1	yes	yes		FALSE	FALSE
POLE	yes	yes	Recurrent	FALSE	FALSE
POLG	yes			FALSE	FALSE
POLQ	yes	yes		FALSE	FALSE
POT1	yes		Dominant	FALSE	FALSE
POU2AF1	yes		Dominant	FALSE	FALSE
POU5F1	yes		Dominant	FALSE	TRUE
PPARG	yes		Dominant	FALSE	FALSE
PPFIBP1	yes		Dominant	FALSE	FALSE
PPM1D	yes		Dominant	FALSE	FALSE
PPP2R1A	yes		Dominant	FALSE	FALSE
PPP6C	yes		Dominant	FALSE	FALSE
PRCC	yes		Dominant	FALSE	TRUE
PRDM1	yes		Recurrent	FALSE	TRUE
PRDM16	yes		Dominant	FALSE	TRUE

© 2020 Morton SU et al. JAMA Cardiology.

Gene Symbol	Somatic	Germline	Molecular Genetics	OMIM CHD Gene	Regulatory Function
PRDM2	yes			FALSE	TRUE
PREX2	yes		Dominant	FALSE	FALSE
PRF1		yes	Recurrent	FALSE	FALSE
PRKACA	yes		Dominant	FALSE	TRUE
PRKAR1A	yes	yes	Dominant/Recurrent	FALSE	FALSE
PRKCB	yes			FALSE	TRUE
PRPF40B	yes			FALSE	TRUE
PRRX1	yes		Dominant	FALSE	TRUE
PSIP1	yes		Dominant	FALSE	TRUE
PTCH1	yes	yes	Recurrent	FALSE	FALSE
PTEN	yes	yes	Dominant	TRUE	FALSE
PTK6	yes		Dominant	FALSE	FALSE
PTPN11	yes		Dominant	TRUE	FALSE
PTPN13	yes	yes	Recurrent	FALSE	FALSE
PTPN6	yes			FALSE	FALSE
PTPRB	yes		Recurrent	FALSE	FALSE
PTPRC	yes		Recurrent	FALSE	FALSE
PTPRD	yes			FALSE	FALSE
PTPRK	yes		Dominant	FALSE	FALSE
PTPRT	yes			FALSE	FALSE
PWWP2A	yes		Dominant	FALSE	FALSE
QKI	yes		Dominant	FALSE	TRUE
RABEP1	yes		Dominant	FALSE	FALSE
RAC1	yes		Dominant	FALSE	FALSE
RAD17	yes			FALSE	FALSE
RAD21	yes		Recurrent	TRUE	FALSE
RAD51B	yes		Dominant	FALSE	FALSE
RAF1	yes		Dominant	TRUE	FALSE
RALGDS	yes		Dominant	FALSE	FALSE
RANBP2	yes		Dominant	FALSE	FALSE
RAP1GDS1	yes		Dominant	FALSE	FALSE
RARA	yes		Dominant	FALSE	TRUE
RB1	yes	yes	Recurrent	FALSE	TRUE
RBM10	yes		Recurrent/X	TRUE	TRUE
RBM15	yes		Dominant	FALSE	TRUE
RECQL4		yes	Recurrent	FALSE	FALSE
REL	yes		Dominant	FALSE	TRUE
RET	yes	yes	Dominant	FALSE	FALSE
RFWD3	yes	yes		FALSE	FALSE
RGPD3	yes			FALSE	FALSE
RGS7	yes			FALSE	FALSE
RHOA	yes		Dominant	FALSE	FALSE
RHOH	yes		Dominant	FALSE	FALSE
RMI2	yes		Dominant	FALSE	FALSE
RNF213	yes		Dominant	FALSE	FALSE
RNF43	yes		Recurrent	FALSE	FALSE
ROBO2	yes			FALSE	FALSE
ROS1	yes		Dominant	FALSE	FALSE
RPL10	yes		Dominant	FALSE	FALSE
RPL22	yes		Dominant	FALSE	FALSE
RPL5	yes		Dominant	TRUE	FALSE
RPN1	yes		Dominant	FALSE	FALSE

© 2020 Morton SU et al. JAMA Cardiology.

Gene Symbol	Somatic	Germline	Molecular Genetics	OMIM CHD Gene	Regulatory Function
RSPO2	yes		Dominant	FALSE	FALSE
RSPO3	yes		Dominant	FALSE	FALSE
RUNDC2A	yes		Dominant	FALSE	FALSE
RUNX1	yes		Dominant	FALSE	TRUE
RUNX1T1	yes		Dominant	FALSE	TRUE
S100A7	yes			FALSE	FALSE
SALL4	yes			FALSE	TRUE
SBDS		yes	Recurrent	FALSE	FALSE
SDC4	yes		Dominant	FALSE	FALSE
SDHA	yes	yes	Recurrent	FALSE	FALSE
SDHAF2		yes	Recurrent	FALSE	FALSE
SDHB		yes	Recurrent	FALSE	FALSE
SDHC		yes	Recurrent	FALSE	FALSE
SDHD		yes	Recurrent	FALSE	FALSE
5-Sep	yes		Dominant	FALSE	FALSE
6-Sep	yes		Dominant	FALSE	FALSE
9-Sep	yes		Dominant	FALSE	FALSE
SET	yes		Dominant	FALSE	FALSE
SETBP1	yes	yes	Dominant	TRUE	TRUE
SETD1B	yes			FALSE	FALSE
SETD2	yes		Recurrent	FALSE	FALSE
SETDB1	yes			FALSE	FALSE
SF3B1	yes		Dominant	FALSE	FALSE
SFPQ	yes		Dominant	FALSE	TRUE
SFRP4	yes		Recurrent	FALSE	FALSE
SGK1	yes			FALSE	FALSE
SH2B3	yes		Recurrent	FALSE	FALSE
SH3GL1	yes		Dominant	FALSE	FALSE
SIRPA	yes			FALSE	FALSE
SIX1	yes			FALSE	TRUE
SIX2	yes			FALSE	TRUE
SKI	yes			TRUE	TRUE
SLC34A2	yes		Dominant	FALSE	FALSE
SLC45A3	yes		Dominant	FALSE	FALSE
SMAD2	yes		Recurrent	FALSE	TRUE
SMAD3	yes		Recurrent	TRUE	TRUE
SMAD4	yes	yes	Recurrent	TRUE	TRUE
SMARCA4	yes		Recurrent	TRUE	TRUE
SMARCB1	yes	yes	Recurrent	TRUE	TRUE
SMARCD1	yes		Recurrent	FALSE	FALSE
SMARCE1		yes	Recurrent	TRUE	TRUE
SMC1A	yes			FALSE	FALSE
SMO	yes		Dominant	FALSE	FALSE
SND1	yes		Dominant	FALSE	FALSE
SOCS1	yes		Recurrent	FALSE	FALSE
SOX2	yes		Dominant	TRUE	TRUE
SOX21	yes			FALSE	TRUE
SPECC1	yes		Dominant	FALSE	FALSE
SPEN	yes		Recurrent	FALSE	TRUE
SPOP	yes	yes	Recurrent	FALSE	FALSE
SRC	yes		Dominant	FALSE	TRUE
SRGAP3	yes		Dominant	FALSE	FALSE

© 2020 Morton SU et al. JAMA Cardiology.

Gene Symbol	Somatic	Germline	Molecular Genetics	OMIM CHD Gene	Regulatory Function
SRSF2	yes		Dominant	FALSE	TRUE
SRSF3	yes		Dominant	FALSE	TRUE
SS18	yes		Dominant	FALSE	FALSE
SS18L1	yes		Dominant	FALSE	FALSE
SSX1	yes		Dominant	FALSE	FALSE
SSX2	yes		Dominant	FALSE	FALSE
SSX4	yes		Dominant	FALSE	FALSE
STAG1	yes			FALSE	FALSE
STAG2	yes		Recurrent	FALSE	FALSE
STAT3	yes	yes	Dominant	FALSE	TRUE
STAT5B	yes		Dominant	FALSE	TRUE
STAT6	yes		Dominant	FALSE	FALSE
STIL	yes		Dominant	FALSE	FALSE
STK11	yes	yes	Recurrent	FALSE	FALSE
STRN	yes		Dominant	FALSE	TRUE
SUFU	yes	yes	Recurrent	FALSE	TRUE
SUZ12	yes		Dominant	FALSE	FALSE
SYK	yes		Dominant	FALSE	FALSE
TAF15	yes		Dominant	FALSE	FALSE
TAL1	yes		Dominant	FALSE	TRUE
TAL2	yes		Dominant	FALSE	TRUE
TBL1XR1	yes		Recurrent	FALSE	FALSE
TBX3	yes		Dominant	TRUE	FALSE
TCEA1	yes		Dominant	FALSE	FALSE
TCF12	yes		Dominant	FALSE	TRUE
TCF3	yes		Dominant	FALSE	TRUE
TCF7L2	yes		Dominant	FALSE	TRUE
TCL1A	yes		Dominant	FALSE	FALSE
TEC	yes			FALSE	FALSE
TERT	yes	yes	Dominant	FALSE	TRUE
TET1	yes		Dominant	FALSE	FALSE
TET2	yes		Recurrent	FALSE	FALSE
TFE3	yes		Dominant	FALSE	TRUE
TFEB	yes		Dominant	FALSE	TRUE
TFG	yes		Dominant	FALSE	FALSE
TFPT	yes		Dominant	FALSE	FALSE
TFRC	yes		Dominant	FALSE	FALSE
TGFBR2	yes	yes	Recurrent	TRUE	FALSE
THRAP3	yes		Dominant	FALSE	TRUE
TLX1	yes		Dominant	FALSE	TRUE
TLX3	yes		Dominant	FALSE	TRUE
TMEM127		yes	Recurrent	FALSE	FALSE
TMPRSS2	yes		Dominant	FALSE	FALSE
TNC	yes			FALSE	FALSE
TNFAIP3	yes		Recurrent	FALSE	FALSE
TNFRSF14	yes		Recurrent	FALSE	FALSE
TNFRSF17	yes		Dominant	FALSE	FALSE
TOP1	yes		Dominant	FALSE	FALSE
TP53	yes	yes	Recurrent	FALSE	TRUE
TP63	yes	yes		FALSE	TRUE
TPM3	yes		Dominant	FALSE	FALSE
TPM4	yes		Dominant	FALSE	FALSE

© 2020 Morton SU et al. JAMA Cardiology.

Gene Symbol	Somatic	Germline	Molecular Genetics	OMIM CHD Gene	Regulatory Function
TPR	yes		Dominant	FALSE	FALSE
TRA	yes		Dominant	FALSE	FALSE
TRAF7	yes		Dominant	FALSE	FALSE
TRB	yes		Dominant	FALSE	FALSE
TRD	yes		Dominant	FALSE	FALSE
TRIM24	yes		Dominant	FALSE	TRUE
TRIM27	yes		Dominant	FALSE	FALSE
TRIM33	yes		Dominant	FALSE	FALSE
TRIP11	yes		Dominant	FALSE	FALSE
TRRAP	yes		Dominant	FALSE	FALSE
TSC1	yes	yes	Recurrent	TRUE	FALSE
TSC2	yes	yes	Recurrent	TRUE	FALSE
TSHR	yes	yes	Dominant	FALSE	FALSE
U2AF1	yes		Dominant	FALSE	TRUE
UBR5	yes		Recurrent	FALSE	FALSE
USP44	yes			FALSE	FALSE
USP6	yes		Dominant	FALSE	FALSE
USP8	yes		Dominant	FALSE	FALSE
VAV1	yes			FALSE	FALSE
VHL	yes	yes	Recurrent	FALSE	TRUE
VTI1A	yes		Dominant	FALSE	FALSE
WAS		yes	Recurrent/X	FALSE	FALSE
WHSC1	yes		Dominant	FALSE	FALSE
WHSC1L1	yes		Dominant	FALSE	FALSE
WIF1	yes		Dominant	FALSE	FALSE
WNK2	yes			FALSE	FALSE
WRN		yes	Recurrent	FALSE	FALSE
WT1	yes	yes	Recurrent	FALSE	TRUE
WWTR1	yes		Dominant	FALSE	FALSE
XPA		yes	Recurrent	FALSE	FALSE
XPC		yes	Recurrent	FALSE	FALSE
XPO1	yes		Dominant	FALSE	FALSE
YWHAE	yes		Dominant	FALSE	FALSE
ZBTB16	yes		Dominant	FALSE	TRUE
ZCCHC8	yes		Dominant	FALSE	TRUE
ZEB1	yes			FALSE	TRUE
ZFHX3	yes		Dominant	FALSE	FALSE
ZMYM3	yes			FALSE	FALSE
ZNF198	yes		Dominant	FALSE	FALSE
ZNF278	yes		Dominant	FALSE	FALSE
ZNF331	yes		Dominant	FALSE	FALSE
ZNF384	yes		Dominant	FALSE	FALSE
ZNF429	yes			FALSE	FALSE
ZNF479	yes			FALSE	FALSE
ZNF521	yes		Dominant	FALSE	TRUE
ZNRF3	yes			FALSE	FALSE
ZRSR2	yes		Recurrent	FALSE	TRUE

© 2020 Morton SU et al. JAMA Cardiology.

eTable 3. Dominant CHD Genes that are also Cancer Risk Genes

Gene Symbol	Somatic Cancer Risk	Germline Cancer Risk	Cancer Syndrome
ARID1A	yes	no	-
ASXL1	yes	no	-
BCOR	yes	no	-
CBL	yes	no	-
COL1A1	yes	no	-
COL2A1	yes	no	-
COL3A1	yes	no	-
CREBBP	yes	no	-
ELN	yes	no	-
FGFR1	yes	no	-
HRAS	yes	yes	Costello syndrome
KAT6B	yes	no	-
KMT2A	yes	no	-
KMT2D	yes	no	-
KRAS	yes	no	-
MAP2K1	yes	no	-
MAP2K2	yes	no	-
NF1	yes	yes	neurofibromatosis type 1
NOTCH1	yes	no	-
NOTCH2	yes	no	-
NRAS	yes	no	-
NSD1	yes	no	-
PTPN11	yes	no	-
RAD21	yes	no	-
RAF1	yes	no	-
RPL5	yes	no	-
SETBP1	yes	yes	Schinzel-Giedion syndrome
SKI	yes	no	-
SMAD3	yes	no	-
SMAD4	yes	yes	juvenile polyposis
SMARCA4	yes		-
SMARCB1	yes	yes	rhabdoid predisposition syndrome
SMARCE1		yes	-
SOX2	yes	no	-
TBX3	yes	no	-
TGFBR2	yes	yes	Hereditary Nonpolyposis Colorectal Cancer
TSC1	yes	yes	Tuberous sclerosis 1
TSC2	yes	yes	Tuberous sclerosis 2

© 2020 Morton SU et al. JAMA Cardiology.

eTable 4. Loss of Function Variants in CHD Discovery and Replication Cohorts

Blinded.ID	Chromosome	Position	Reference	Alternate	Variant Effect	Gene	Cohort*
1-03179	1	6931821	C	G	STOP_GAINED	CAMTA1	D
1-04241	1	7805023	G	A	START_LOST	CAMTA1	R
1-03444	1	11301639	CA	C	FRAME_SHIFT	MTOR	R
1-02173	1	11301740	T	C	SPLICE_ACCEPTOR_VARIANT&INTR	MTOR	R
1-05588	1	14142944	C	T	STOP_GAINED	PRDM2	D
1-12378	1	15844830	G	A	STOPGAIN	CASP9	R
1-09417	1	36931786	G	A	STOP_GAINED	CSF3R	D
1-04934	1	36932914	T	G	SPLICE_SITE_ACCEPTOR	CSF3R	D
1-04247	1	43804307	CT	C	FRAMESHIFT_VARIANT	MPL	R
1-16712	1	45798843	C	G	SPLICE_ACCEPTOR_VARIANT&INTR	MUTYH	D
1-10477	1	47685569	G	GCCCCCCCCCCCCC	FRAMESHIFT_VARIANT	TAL1	D
S28W1F45	1	47685569	G	GCCCCCCCCCCCCC	FRAMESHIFT_VARIANT	TAL1	D
1-00953	1	47685569	G	GCCCCCCCCCCCCC	FRAMESHIFT_VARIANT	TAL1	D
S7D273FN	1	47685569	G	GCCCCCCCCCCCCC	FRAMESHIFT_VARIANT	TAL1	R
1-02176	1	47685569	GC	G	FRAMESHIFT_VARIANT	TAL1	R
1-08730	1	47767349	T	TC	FRAMESHIFT_INSERTION	STIL	R
1-06321	1	51871728	AACTG	A	FRAMESHIFT_DELETION	EPS15	R
1-02988	1	51871782	T	C	SPLICE_SITE_ACCEPTOR	EPS15	D
1-05496	1	51873982	A	AAT	FRAMESHIFT_INSERTION	EPS15	D
1-08084	1	93299009	C	T	STOP_GAINED	RPL5	R
1-09781	1	93299101	G	C	SPLICE_SITE_ACCEPTOR	RPL5	D
1-00726	1	93299193	GACAA	G	FRAMESHIFT_DELETION	RPL5	D
1-02338	1	120468139	CT	C	FRAMESHIFT_DELETION	NOTCH2	R
1-07308	1	120484379	T	C	SPLICE_SITE	NOTCH2	D
1-01577	1	120491190	C	A	SPLICE_SITE	NOTCH2	D
1-06409	1	144922191	AG	A	FRAMESHIFT_DELETION	PDE4DIP	D
1-03652	1	144922259	TAC	T	FRAMESHIFT_DELETION	PDE4DIP	D
1-02123	1	144922572	G	A	STOPGAIN	PDE4DIP	D
1-03623	1	144922617	C	A	STOPGAIN	PDE4DIP	D
1-07149	1	147096239	C	T	STOPGAIN	BCL9	D
1-16320	1	155159827	C	CT	FRAMESHIFT_VARIANT	MUC1	D
SP58I8VB	1	155160228	G	A	STOP_GAINED	MUC1	D
1-15748	1	155160228	G	A	STOP_GAINED	MUC1	R
1-05536	1	155160228	G	A	STOPGAIN	MUC1	R
1-04659	1	155160740	AG	A	FRAMESHIFT_DELETION	MUC1	R
GT04006381	1	155162578	TG	T	FRAMESHIFT_DELETION	MUC1	D
1-04380	1	156830932	CTG	C	FRAMESHIFT_DELETION	NTRK1	D
1-05567	1	179077575	GA	G	FRAMESHIFT_DELETION	ABL2	D
1-05105	1	179077575	GA	G	FRAMESHIFT_DELETION	ABL2	R
1-02674	1	179084038	A	AG	FRAMESHIFT_INSERTION	ABL2	R
1-06369	1	179086615	GT	G	FRAMESHIFT_VARIANT	ABL2	D
1-01468	1	186283116	CCT	C	FRAMESHIFT_DELETION	TPR	R
1-01445	1	193111145	CAGAG	C	FRAMESHIFT_DELETION	CDC73	R
1-05068	1	201984437	C	T	STOPGAIN	ELF3	D
1-01953	1	241099899	C	T	SPLICE_DONOR_VARIANT&INTRON	RGS7	R
1-06241	1	241669455	TA	T	FRAMESHIFT_DELETION	FH	R
1-02377	1	241682982	A	AG	FRAMESHIFT_INSERTION	FH	D
1-15195	2	25457149	TAC	T	FRAMESHIFT_VARIANT&STOP_LOS	DNMT3A	R
1-07108	2	25964922	GCA	G	FRAMESHIFT_DELETION	ASXL2	D
1-02496	2	29446207	C	A	SPLICE_SITE	ALK	R
1-01364	2	29606630	AC	A	FRAMESHIFT_DELETION	ALK	R
1-02860	2	30143070	CTCCAAGA	C	FRAME_SHIFT	ALK	R
1-01638	2	30143364	CAG	C	FRAMESHIFT_DELETION	ALK	D
1-07443	2	42513501	C	A	STOP_GAINED	EML4	R

© 2020 Morton SU et al. JAMA Cardiology.

Blinded.ID	Chromosome	Position	Reference	Alternate	Variant Effect	Gene	Cohort*
1-12248	2	42515464	T	C	SPLICE_SITE_ACCEPTOR	EML4	D
1-02121	2	46603838	G	T	STOPGAIN	EPAS1	D
1-06225	2	46605874	GAC	G	FRAMESHIFT_DELETION	EPAS1	D
1-07464	2	47702239	C	CA	FRAMESHIFT_INSERTION	MSH2	R
1-03590	2	48030604	CTA	C	FRAMESHIFT_DELETION	MSH6	D
1-17672	2	48033736	G	GACATAGAAAA	FRAMESHIFT_VARIANT&STOP_GAI	MSH6	R
1-01963	2	74594223	A	AC	FRAMESHIFT_INSERTION	DCTN1	D
SMVB8866	2	100209822	C	T	STOP_GAINED	AFF3	D
1-07619	2	113993081	C	G	SPLICE_ACCEPTOR_VARIANT&INTR	PAX8	D
1-02885	2	128029024	AC	A	FRAMESHIFT_DELETION	ERCC3	D
1-14872	2	128038034	G	A	STOP_GAINED	ERCC3	R
SYQSHY52	2	141115547	C	T	SPLICE_DONOR_VARIANT&INTRON	LRP1B	D
1-08490	2	141806571	TTCTC	T	FRAMESHIFT_DELETION	LRP1B	R
1-04188	2	158594140	C	T	STOPGAIN	ACVR1	R
1-06664	2	178095583	AG	A	FRAMESHIFT_DELETION	NFE2L2	R
SPER4I68	2	187490314	C	T	STOP_GAINED&SPLICE_REGION_V/	ITGAV	D
1-06404	2	187521027	C	T	STOPGAIN	ITGAV	R
1-07737	2	187534481	G	GC	FRAMESHIFT_INSERTION	ITGAV	D
1-02758	2	189861194	TG	T	FRAMESHIFT_DELETION	COL3A1	D
1-01880	2	189864084	GT	G	FRAMESHIFT_DELETION	COL3A1	D
S66B4E3U	2	190719427	G	T	STOP_GAINED	PMS1	D
1-02823	2	190719562	A	T	STOPGAIN	PMS1	R
1-12549	2	190728665	C	T	STOP_GAINED	PMS1	R
1-00166	2	198283314	T	C	SPLICE_SITE_ACCEPTOR	SF3B1	D
1-09874	2	202131488	AGGCAG	A	FRAMESHIFT_VARIANT	CASP8	R
1-06459	2	202134311	CG	C	FRAMESHIFT_DELETION	CASP8	R
1-13229	2	202150030	C	T	STOPGAIN	CASP8	R
1-02020	2	209103892	CA	C	FRAMESHIFT_DELETION	IDH1	D
1-01845	2	209113209	G	A	STOPGAIN	IDH1	R
1-03915	2	215646150	G	A	STOPGAIN	BARD1	R
1-02680	2	215657091	AT	A	FRAMESHIFT_DELETION	BARD1	R
S5NR2EQD	2	215661783	A	G	SPLICE_DONOR_VARIANT&INTRON	BARD1	R
1-13512	2	216197212	A	T	STOP_GAINED	ATIC	D
1-01038	2	223065913	C	A	STOPGAIN	PAX3	D
S75UT2U8	2	223158842	C	T	STOP_GAINED	PAX3	R
1-05911	2	225365137	GCTGA	G	FRAMESHIFT_DELETION	CUL3	D
1-07184	2	225379474	G	A	STOP_GAINED	CUL3	D
1-07320	3	10094155	CAG	C	FRAMESHIFT_DELETION	FANCD2	D
1-03385	3	10191647	TGAA	T	FRAMESHIFT_DELETION	VHL	R
1-13470	3	13663316	C	T	STOP_GAINED	FBLN2	R
1-01477	3	14200140	G	A	STOPGAIN	XPC	D
1-01451	3	14200140	G	A	STOPGAIN	XPC	R
1-05066	3	14208722	GAT	G	FRAMESHIFT_VARIANT	XPC	D
1-01952	3	30729962	C	T	STOPGAIN	TGFBR2	R
1-01221	3	37038201	G	T	SPLICE_SITE	MLH1	D
1-04805	3	37050304	G	C	SPLICE_SITE	MLH1	D
1-02828	3	41277216	AG	A	FRAME_SHIFT	CTNNB1	D
1-15566	3	41277241	T	TG	FRAMESHIFT_VARIANT	CTNNB1	R
1-15566	3	41277241	T	TG	FRAMESHIFT_VARIANT	CTNNB1	R
1-06579	3	41280659	T	G	STOP_GAINED	CTNNB1	R
1-02881	3	48712024	G	A	STOPGAIN	NCKIPSD	R
GT04006992	3	48716340	C	A	STOPGAIN	NCKIPSD	R
1-02090	3	48717140	G	A	STOPGAIN	NCKIPSD	D
SD72ZZSN	3	48719511	G	A	STOP_GAINED	NCKIPSD	D

© 2020 Morton SU et al. JAMA Cardiology.

Blinded.ID	Chromosome	Position	Reference	Alternate	Variant Effect	Gene	Cohort*
1-04924	3	49397734	T	A	STOPGAIN	RHOA	R
1-02468	3	52441334	T	TG	SPLICE_SITE	BAP1	R
1-08561	3	71179746	GGGGTTGCC	G	FRAMESHIFT_DELETION	FOXP1	R
1-08615	3	105397416	C	T	SPLICE_ACCEPTOR_VARIANT&INTR	CBLB	R
1-01552	3	105400456	T	C	SPLICE_SITE_ACCEPTOR	CBLB	R
1-04158	3	105495261	C	T	STOPGAIN	CBLB	D
SA53ZGO2	3	121151265	C	G	SPLICE_ACCEPTOR_VARIANT&INTR	POLQ	R
GT04011042	3	121179001	G	A	STOPGAIN	POLQ	R
1-02492	3	121179030	GC	G	FRAMESHIFT_DELETION	POLQ	D
1-01457	3	121190878	AT	A	FRAMESHIFT_DELETION	POLQ	D
1-02671	3	121202430	CT	C	SPLICE_SITE	POLQ	R
1-04724	3	121207197	A	T	STOPGAIN	POLQ	R
1-04162	3	121207489	A	T	STOP_GAINED	POLQ	D
1-16205	3	121207497	AC	A	FRAMESHIFT_VARIANT	POLQ	D
SPER4I68	3	121208129	GTCTC	G	FRAMESHIFT_VARIANT	POLQ	D
1-08832	3	121208378	C	CA	FRAMESHIFT_INSERTION	POLQ	D
1-00181	3	121208923	G	C	STOPGAIN	POLQ	D
1-02920	3	121209036	AC	A	FRAMESHIFT_DELETION	POLQ	D
1-02963	3	121212372	AT	A	FRAMESHIFT_DELETION	POLQ	R
1-04297	3	136096595	C	G	SPLICE_ACCEPTOR_VARIANT&INTR	STAG1	R
1-12251	3	138461576	G	A	STOP_GAINED	PIK3CB	R
1-03707	3	142234358	CT	C	SPLICE_SITE	ATR	R
SNO4V75I	3	142281489	A	T	STOP_GAINED	ATR	D
1-06372	3	149374854	C	CCCAG	FRAME_SHIFT	WWTR1	R
1-06642	3	158315983	C	T	STOPGAIN	MLF1	D
1-07728	3	185538826	G	GTAGT	STOPGAIN	IGF2BP2	D
1-02185	3	186504953	CACTG	C	FRAME_SHIFT	EIF4A2	D
1-04246	3	188202383	A	AT	FRAMESHIFT_INSERTION	LPP	R
1-15553	3	188242554	CT	C	FRAMESHIFT_VARIANT	LPP	R
1-00719	3	188327066	C	T	STOP_GAINED	LPP	D
1-03432	3	192516543	CCAGA	C	FRAMESHIFT_DELETION	MB21D2	D
1-04046	3	195488364	C	A	STOPGAIN	MUC4	R
1-12565	3	195492306	GC	G	FRAMESHIFT_DELETION	MUC4	D
1-01715	3	195515653	G	C	STOPGAIN	MUC4	D
1-04920	3	195778948	CA	C	STOPGAIN	TFRC	R
1-02054	3	195789728	T	TA	FRAMESHIFT_INSERTION	TFRC	R
1-06165	3	195798350	TG	T	FRAMESHIFT_DELETION	TFRC	R
1-00290	4	1940175	TAGAG	T	FRAME_SHIFT	WHSC1	R
S13JG491	4	48151696	G	T	STOP_GAINED	TEC	D
1-06302	4	48170609	TGTTTCTG	T	FRAMESHIFT_VARIANT	TEC	R
1-01405	4	54245238	A	G	SPLICE_SITE	FIP1L1	D
1-05359	4	54280858	C	T	STOPGAIN	FIP1L1	D
1-00872	4	55139837	C	T	STOPGAIN	PDGFRA	D
S389MD2B	4	55955618	T	TAA	FRAMESHIFT_VARIANT	KDR	D
SP58I8VB	4	55956247	T	C	SPLICE_ACCEPTOR_VARIANT&INTR	KDR	D
S6C9ENR7	4	55956247	T	C	SPLICE_ACCEPTOR_VARIANT&INTR	KDR	R
1-12463	4	55968574	G	A	STOP_GAINED	KDR	R
1-07439	4	55971153	T	A	SPLICE_SITE	KDR	R
1-07375	4	55972059	T	A	STOPGAIN	KDR	D
SOHDWSV6	4	55979645	G	A	STOP_GAINED	KDR	D
SRS4AH92	4	87516940	G	A	SPLICE_DONOR_VARIANT&INTRON	PTPN13	R
1-04555	4	87671811	C	T	STOPGAIN	PTPN13	R
1-05190	4	87692532	C	A	STOPGAIN	PTPN13	D
1-01309	4	87696660	A	AGG	FRAMESHIFT_INSERTION	PTPN13	R

© 2020 Morton SU et al. JAMA Cardiology.

Blinded.ID	Chromosome	Position	Reference	Alternate	Variant Effect	Gene	Cohort*
1-01577	4	87720325	TAGAG	T	FRAMESHIFT_DELETION	PTPN13	D
S7943779	4	87968660	C	T	STOP_GAINED	AFF1	R
1-00437	4	88055830	C	CA	FRAMESHIFT_INSERTION	AFF1	R
1-00884	4	108984821	T	C	SPLICE_SITE_DONOR	LEF1	D
1-16164	4	153273675	T	TC	FRAMESHIFT_VARIANT	FBXW7	D
1-02773	4	187518186	A	G	SPLICE_DONOR_VARIANT&INTRON	FAT1	R
1-00526	4	187518911	CA	C	FRAMESHIFT_VARIANT	FAT1	R
1-09064	4	187540074	G	A	STOPGAIN	FAT1	R
1-06077	4	187584530	G	T	STOPGAIN	FAT1	R
1-00101	5	31515588	C	T	STOPGAIN	DROSHA	R
GT04010822	5	31521294	G	A	STOPGAIN	DROSHA	D
1-14135	5	35874632	T	A	STOPGAIN	IL7R	R
SWX1WC3T	5	38506118	G	A	STOP_GAINED	LIFR	D
1-01412	5	38510673	T	TC	FRAMESHIFT_INSERTION	LIFR	D
1-00515	5	38511915	C	T	STOPGAIN	LIFR	D
1-14009	5	55247868	CT	C	FRAMESHIFT_VARIANT	IL6ST	D
1-06822	5	56189498	T	TA	FRAMESHIFT_INSERTION	MAP3K1	D
SN328K5N	5	68692332	C	T	STOP_GAINED	RAD17	D
1-03859	5	112179280	CTG	C	FRAMESHIFT_DELETION	APC	D
1-03160	5	131326601	G	T	STOP_GAINED	ACSL6	R
S1A8DTH5	5	131347217	GC	G	FRAMESHIFT_VARIANT	ACSL6	D
SK31QQSO	5	131347217	GC	G	FRAMESHIFT_VARIANT	ACSL6	R
1-05486	5	149782175	C	T	STOPGAIN	CD74	D
1-07533	5	156671318	G	T	STOP_GAINED	ITK	D
1-05414	5	170837531	G	GTTTTT	FRAMESHIFT_VARIANT&SPLICE_RE	NPM1	D
1-00949	5	176517937	A	C	SPLICE_SITE	FGFR4	D
1-03150	5	176520416	G	A	STOPGAIN	FGFR4	R
1-07985	5	176523702	G	T	STOPGAIN	FGFR4	D
1-12252	5	176673717	C	T	STOP_GAINED	NSD1	R
1-01904	5	176709538	C	T	STOP_GAINED	NSD1	R
1-15195	5	176710791	C	T	STOP_GAINED	NSD1	R
1-15195	5	176710791	C	T	STOP_GAINED	NSD1	R
1-02563	5	176722213	AGAAGT	A	FRAME_SHIFT	NSD1	R
1-09295	5	180038338	CCAGGCTGTG	C	FRAMESHIFT_VARIANT	FLT4	D
1-04533	5	180045776	G	A	STOPGAIN	FLT4	D
1-03825	5	180046025	CAG	C	STOP_GAINED	FLT4	R
1-06642	5	180046066	GA	G	FRAMESHIFT_DELETION	FLT4	D
SC72XCU6	5	180047172	C	T	SPLICE_DONOR_VARIANT&INTRON	FLT4	D
1-00645	5	180047969	G	A	STOPGAIN	FLT4	R
S9KQ7CBT	5	180055882	C	T	STOP_GAINED&SPLICE_REGION_V/	FLT4	D
1-01795	5	180055896	C	CG	FRAMESHIFT_INSERTION	FLT4	R
1-04970	5	180055902	G	C	STOPGAIN	FLT4	D
1-00788	5	180057231	CAGCG	C	FRAMESHIFT_DELETION	FLT4	D
1-11643	5	180057650	TGTACCTC	T	FRAMESHIFT_VARIANT	FLT4	R
1-14281	5	180057711	G	A	STOPGAIN	FLT4	D
1-03410	5	180058747	CG	C	FRAME_SHIFT	FLT4	D
1-05967	5	180058747	CG	C	FRAMESHIFT_DELETION	FLT4	D
SPM8STMK	5	180058747	C	CG	FRAMESHIFT_VARIANT	FLT4	R
1-07373	6	405076	CT	C	FRAMESHIFT_DELETION	IRF4	D
1-05357	6	27107119	TG	T	FRAMESHIFT_DELETION	HIST1H4I	R
1-17141	6	35420324	T	A	START_LOST	FANCE	R
1-02173	6	35430683	C	T	STOP_GAINED	FANCE	R
1-01045	6	36652057	AG	A	FRAMESHIFT_DELETION	CDKN1A	R
1-04570	6	36652219	C	G	STOP_GAINED	CDKN1A	R

© 2020 Morton SU et al. JAMA Cardiology.

Blinded.ID	Chromosome	Position	Reference	Alternate	Variant Effect	Gene	Cohort*
1-01169	6	55623840	TG	T	FRAMESHIFT_DELETION	BMP5	D
1-01767	6	94129046	GT	G	FRAME_SHIFT	EPHA7	R
1-03887	6	99992986	A	T	SPLICE_DONOR_VARIANT&INTRON	CCNC	R
1-03887	6	99992987	C	CA	FRAMESHIFT_VARIANT&SPLICE_RE	CCNC	R
1-15371	6	106555153	AG	A	FRAMESHIFT_VARIANT	PRDM1	R
1-03254	6	106555336	A	AC	FRAMESHIFT_INSERTION	PRDM1	R
1-03360	6	117715824	C	A	STOP_GAINED	ROS1	R
1-12875	6	117718127	G	A	STOPGAIN	ROS1	D
1-04270	6	117724380	G	A	STOPGAIN	ROS1	D
1-03279	6	117725481	T	A	STOPGAIN	ROS1	D
1-07737	6	117739633	TCA	T	FRAMESHIFT_DELETION	ROS1	D
1-06204	6	117888017	C	A	STOPGAIN	GOPC	R
1-06753	6	127516992	G	GA	FRAMESHIFT_INSERTION	RSPO3	D
GT04008062	6	127516992	G	GA	FRAMESHIFT_INSERTION	RSPO3	R
1-06070	6	128302304	A	AT	FRAMESHIFT_INSERTION	PTPRK	D
1-05682	6	128326313	G	A	STOPGAIN	PTPRK	D
1-01805	6	128388900	G	A	STOPGAIN	PTPRK	R
1-08646	6	139165635	C	T	STOP_GAINED	ECT2L	D
1-01253	6	139186258	A	T	STOPGAIN	ECT2L	D
GT04006181	6	152415579	C	T	STOPGAIN	ESR1	D
1-04638	6	157469898	C	T	STOP_GAINED	ARID1B	R
1-04000	6	167435938	C	CT	FRAMESHIFT_INSERTION	FGFR1OP	R
1-03413	6	167453464	T	C	STOPLOSS	FGFR1OP	D
1-00524	6	168289992	G	A	STOPGAIN	MLLT4	D
1-05752	6	168311774	C	CT	SPLICE_ACCEPTOR_VARIANT&INTR	MLLT4	R
1-05588	6	168351926	C	T	STOP_GAINED	MLLT4	D
1-04570	7	2966372	C	G	SPLICE_DONOR_VARIANT&INTRON	CARD11	R
1-00874	7	6029461	C	CTA	STOPGAIN	PMS2	D
1-15672	7	6038900	CA	C	FRAMESHIFT_VARIANT	PMS2	R
1-03467	7	14025801	T	C	START_LOST	ETV1	R
1-15028	7	14028639	G	GA	FRAMESHIFT_VARIANT	ETV1	R
1-07502	7	20193959	G	A	STOPGAIN	MACC1	D
S28W1F45	7	20198285	CA	C	FRAMESHIFT_VARIANT	MACC1	D
1-02233	7	20198816	CTG	C	FRAMESHIFT_DELETION	MACC1	R
1-05084	7	20199103	GC	G	FRAMESHIFT_DELETION	MACC1	D
1-10611	7	20199680	GA	G	FRAMESHIFT_VARIANT	MACC1	D
1-06170	7	55240815	G	T	STOPGAIN	EGFR	D
1-07027	7	73458251	G	A	SPLICE_SITE	ELN	R
1-13229	7	73461030	TG	T	FRAME_SHIFT	ELN	R
1-04353	7	73474205	CA	C	FRAMESHIFT_DELETION	ELN	R
1-06582	7	73474514	C	T	STOP_GAINED	ELN	D
1-00077	7	73474832	G	A	SPLICE_DONOR_VARIANT&INTRON	ELN	R
1-03905	7	73474880	GA	G	FRAMESHIFT_DELETION	ELN	R
1-05211	7	73475454	A	T	STOPGAIN	ELN	D
1-02958	7	73480308	C	CG	FRAMESHIFT_INSERTION	ELN	R
STH7C91S	7	86479790	G	T	STOP_GAINED	GRM3	D
1-08795	7	91603037	C	T	STOP_GAINED	AKAP9	D
1-10549	7	91603037	C	T	STOP_GAINED	AKAP9	D
1-00051	7	91630202	TA	T	FRAMESHIFT_DELETION	AKAP9	D
1-04623	7	91631428	CAA	C	FRAMESHIFT_VARIANT	AKAP9	D
1-00226	7	91631777	CT	C	FRAMESHIFT_DELETION	AKAP9	R
1-04061	7	91691722	C	T	STOPGAIN	AKAP9	R
1-00697	7	91700294	C	T	STOPGAIN	AKAP9	D
1-01068	7	98552928	G	T	SPLICE_DONOR_VARIANT&INTRON	TRRAP	R

© 2020 Morton SU et al. JAMA Cardiology.

Blinded.ID	Chromosome	Position	Reference	Alternate	Variant Effect	Gene	Cohort*
1-01954	7	101840013	A	AG	FRAMESHIFT_VARIANT	CUX1	D
1-01708	7	101918631	G	GT	SPLICE_SITE_DONOR	CUX1	D
1-01915	7	116340049	GA	G	FRAMESHIFT_DELETION	MET	D
1-05336	7	124487046	C	CCA	FRAMESHIFT_INSERTION	POT1	D
1-02621	7	128846121	C	T	STOPGAIN	SMO	R
S89OU81B	7	128852218	CAG	C	FRAMESHIFT_VARIANT	SMO	R
SQIHG4NR	7	137597606	CAG	C	FRAMESHIFT_VARIANT	CREB3L2	R
1-05551	7	145814066	G	T	SPLICE_SITE	CNTNAP2	R
1-05092	7	146536997	G	C	SPLICE_SITE	CNTNAP2	D
1-12941	7	146805238	G	T	SPLICE_SITE_ACCEPTOR	CNTNAP2	D
SFY9KO72	7	147815325	G	A	STOP_GAINED	CNTNAP2	D
1-05153	7	151893097	CTAAA	C	SPLICE_SITE	KMT2C	R
1-04307	7	151919733	C	A	STOP_GAINED	KMT2C	D
1-00206	8	1846623	C	T	STOPGAIN	ARHGEF10	D
1-11777	8	1851619	T	C	SPLICE_DONOR_VARIANT&INTRON	ARHGEF10	R
1-09658	8	1853883	G	GT	SPLICE_SITE_DONOR	ARHGEF10	D
1-01457	8	17817937	C	CA	FRAMESHIFT_INSERTION	PCM1	D
1-05393	8	17823962	G	A	STOPGAIN	PCM1	D
1-05678	8	30922560	CAG	C	FRAMESHIFT_DELETION	WRN	R
1-07175	8	30938491	CTTATT	C	FRAMESHIFT_DELETION	WRN	R
1-06483	8	30982140	G	C	SPLICE_SITE	WRN	D
1-07031	8	31012272	G	GT	SPLICE_SITE	WRN	D
1-03143	8	32406296	C	T	STOP_GAINED	NRG1	D
1-16478	8	32617902	C	T	STOP_GAINED	NRG1	D
1-04215	8	41791712	CT	C	FRAME_SHIFT	KAT6A	D
1-00876	8	57079448	A	T	STOPGAIN	PLAG1	D
1-12289	8	68931826	G	T	STOPGAIN	PREX2	D
1-01955	8	69136793	G	A	SPLICE_SITE	PREX2	D
S2LEPW3R	8	69143602	G	GGA	FRAMESHIFT_VARIANT	PREX2	D
S6Y7BAAP	8	69143602	G	GGA	FRAMESHIFT_VARIANT	PREX2	D
1-11533	8	88218367	G	A	SPLICE_DONOR_VARIANT&INTRON	CNBD1	R
1-06330	8	90990546	T	TA	FRAMESHIFT_INSERTION	NBN	R
1-05029	8	90992982	A	AT	FRAMESHIFT_INSERTION	NBN	D
1-04194	8	93088243	CTCAGTGCTC	C	FRAMESHIFT_DELETION	RUNX1T1	R
1-11377	8	95158235	GTGCTGATCA	G	FRAMESHIFT_VARIANT	CDH17	D
1-03700	8	109001398	G	A	STOPGAIN	RSPO2	R
1-09597	8	113293429	C	T	STOP_GAINED	CSMD3	R
1-09597	8	113293429	C	T	STOP_GAINED	CSMD3	R
1-04603	8	113299477	T	C	SPLICE_SITE_ACCEPTOR	CSMD3	D
1-01441	8	113347693	CT	C	FRAMESHIFT_DELETION	CSMD3	R
1-04422	8	113678525	TAG	T	FRAMESHIFT_DELETION	CSMD3	R
1-13938	8	113697632	T	TA	SPLICE_DONOR_VARIANT&INTRON	CSMD3	D
SSGLC3NS	8	113697632	T	TA	SPLICE_DONOR_VARIANT&INTRON	CSMD3	R
1-06857	8	113988131	CT	C	FRAMESHIFT_VARIANT	CSMD3	D
SS2BKWLF	8	117864225	G	A	STOP_GAINED	RAD21	D
S558IAN3	8	117875443	TA	T	FRAMESHIFT_VARIANT	RAD21	R
1-07442	8	118830682	C	A	STOPGAIN	EXT1	D
1-13022	8	128748844	A	AT	FRAMESHIFT_INSERTION	MYC	D
1-07518	8	134260166	G	T	STOP_GAINED	NDRG1	R
1-07305	8	134262739	GT	G	FRAMESHIFT_DELETION	NDRG1	D
S3XYE6KG	8	134271467	CAT	C	FRAMESHIFT_VARIANT	NDRG1	R
1-00619	8	145738323	G	A	STOPGAIN	RECQL4	D
SJ1DO63R	8	145738446	G	A	STOP_GAINED	RECQL4	R
1-08575	8	145741648	TG	T	FRAMESHIFT_DELETION	RECQL4	R

© 2020 Morton SU et al. JAMA Cardiology.

Blinded.ID	Chromosome	Position	Reference	Alternate	Variant Effect	Gene	Cohort*
1-10288	9	8500829	T	TC	FRAMESHIFT_VARIANT	PTPRD	D
1-05572	9	15468820	GACTA	G	FRAMESHIFT_DELETION	PSIP1	D
1-09857	9	35074148	AG	A	FRAMESHIFT_VARIANT	FANCG	R
1-07621	9	35079239	C	A	SPLICE_SITE	FANCG	D
1-07037	9	97888864	C	G	SPLICE_SITE	FANCC	R
1-09032	9	98278970	C	A	STOPGAIN	PTCH1	D
1-03837	9	117808853	A	AT	FRAMESHIFT_INSERTION	TNC	D
1-05783	9	117808853	A	AT	FRAMESHIFT_VARIANT	TNC	R
1-05684	9	117826243	G	A	STOPGAIN	TNC	R
1-09757	9	117827033	CGAAGGCT	C	FRAMESHIFT_DELETION	TNC	D
1-00281	9	117836018	C	T	STOPGAIN	TNC	D
STYKO5EC	9	123857289	A	T	STOP_GAINED	CNTRL	D
1-02974	9	123860663	G	T	SPLICE_SITE	CNTRL	R
S71LMUQ1	9	123900821	A	G	SPLICE_ACCEPTOR_VARIANT&INTR	CNTRL	R
S27W9514	9	123900958	G	A	SPLICE_DONOR_VARIANT&INTRON	CNTRL	R
GT04007271	9	123907545	AG	A	FRAMESHIFT_DELETION	CNTRL	D
1-01861	9	123912579	GC	G	FRAMESHIFT_DELETION	CNTRL	R
SI9F79RQ	9	123919749	C	CA	FRAMESHIFT_VARIANT	CNTRL	R
1-02735	9	123930548	C	T	STOPGAIN	CNTRL	D
1-02467	9	134011379	C	T	STOPGAIN	NUP214	D
S433D2J5	9	134073820	C	T	STOP_GAINED	NUP214	D
1-16378	9	134098187	CAG	C	FRAMESHIFT_VARIANT	NUP214	D
1-05915	9	135781073	GCTTT	G	FRAME_SHIFT	TSC1	D
S677151X	9	136004559	C	T	STOP_GAINED	RALGDS	D
1-13533	9	139396487	C	T	STOP_GAINED	NOTCH1	R
1-13533	9	139396487	C	T	STOP_GAINED	NOTCH1	R
1-02041	9	139399785	G	T	STOPGAIN	NOTCH1	R
1-12329	9	139403438	G	A	STOPGAIN	NOTCH1	R
1-04002	9	139408961	C	A	SPLICE_SITE_DONOR	NOTCH1	R
1-00692	9	139410036	T	TCG	FRAMESHIFT_INSERTION	NOTCH1	R
S4WIFQ51	9	139410107	G	T	STOP_GAINED	NOTCH1	D
1-01026	9	139411749	A	AC	FRAMESHIFT_INSERTION	NOTCH1	R
1-06933	9	139412303	G	A	STOPGAIN	NOTCH1	R
S1A8DTH5	9	139412348	G	GCACA	FRAMESHIFT_VARIANT	NOTCH1	D
SK31QQSO	9	139412348	G	GCACA	FRAMESHIFT_VARIANT	NOTCH1	R
1-06774	9	139417399	G	T	STOP_GAINED	NOTCH1	R
1-00305	9	139418294	AAGCCC	A	FRAMESHIFT_DELETION	NOTCH1	D
1-00953	10	21962451	A	AG	FRAMESHIFT_VARIANT	MLLT10	D
SM5ZY45C	10	22016845	GTC	G	FRAMESHIFT_VARIANT	MLLT10	D
1-06197	10	22029082	G	A	SPLICE_ACCEPTOR_VARIANT&INTR	MLLT10	R
1-02675	10	27066140	G	A	STOP_GAINED	ABI1	D
1-14281	10	31815767	TC	T	FRAMESHIFT_DELETION	ZEB1	D
1-07688	10	32307029	A	AT	FRAMESHIFT_INSERTION	KIF5B	R
1-04812	10	32326210	TAA	T	FRAMESHIFT_DELETION	KIF5B	D
1-06857	10	70405724	C	T	STOP_GAINED	TET1	D
1-01404	10	70426835	CTTTG	C	FRAMESHIFT_DELETION	TET1	R
1-14519	10	76741660	C	T	STOP_GAINED	KAT6B	R
1-07058	10	76788475	G	GA	FRAME_SHIFT	KAT6B	R
1-02925	10	89653789	T	G	STOP_GAINED	PTEN	R
1-03139	10	89711972	AGAT	A	FRAMESHIFT_DELETION	PTEN	D
1-00459	10	89717718	C	CTG	FRAME_SHIFT	PTEN	D
1-04247	10	96797013	GA	G	FRAMESHIFT_VARIANT	CYP2C8	R
S4A8Q7HY	10	96827444	G	C	STOP_GAINED	CYP2C8	D
1-01966	10	114298023	AAC	A	FRAMESHIFT_DELETION	VTI1A	R

© 2020 Morton SU et al. JAMA Cardiology.

Blinded.ID	Chromosome	Position	Reference	Alternate	Variant Effect	Gene	Cohort*
1-06499	10	114428033	C	T	STOPGAIN	VTI1A	R
1-06342	10	131506214	C	T	STOP_GAINED	MGMT	R
1-01242	11	532677	TC	T	FRAMESHIFT_DELETION	HRAS	D
1-02107	11	22646701	TGG	T	FRAMESHIFT_DELETION	FANCF	R
1-01848	11	33886263	G	A	STOPGAIN	LMO2	D
1-02761	11	33891013	C	CCTCGGGTG	FRAMESHIFT_INSERTION	LMO2	D
1-04019	11	44255794	G	T	SPLICE_SITE_DONOR	EXT2	D
1-06642	11	44255794	G	T	SPLICE_SITE_DONOR	EXT2	D
1-03982	11	44255795	T	C	SPLICE_SITE	EXT2	D
1-06481	11	47236741	C	CCCCAGGAA	FRAMESHIFT_VARIANT	DDB2	D
1-06976	11	47259427	C	T	STOPGAIN	DDB2	R
1-15786	11	57559115	G	A	START_LOST	CTNND1	R
1-02956	11	61205585	G	T	STOPGAIN	SDHAF2	R
1-05179	11	61213486	CAA	C	FRAMESHIFT_DELETION	SDHAF2	R
1-04719	11	71721846	G	A	STOPGAIN	NUMA1	R
1-06814	11	71725647	G	A	STOP_GAINED	NUMA1	D
1-01556	11	92086487	AC	A	FRAMESHIFT_DELETION	FAT3	R
1-10826	11	92539641	TG	T	FRAMESHIFT_VARIANT	FAT3	R
1-08441	11	92623805	CAT	C	FRAMESHIFT_VARIANT	FAT3	R
1-08058	11	95826474	G	A	STOPGAIN	MAML2	D
SXTE51B7	11	102195377	C	G	STOP_GAINED	BIRC3	R
1-04692	11	102195819	C	CT	FRAMESHIFT_INSERTION	BIRC3	D
1-03099	11	102197493	C	A	STOP_GAINED	BIRC3	R
1-10612	11	108106449	GA	G	FRAMESHIFT_VARIANT	ATM	R
1-13288	11	108119750	AG	A	FRAMESHIFT_VARIANT	ATM	R
1-05123	11	108124753	AT	A	FRAMESHIFT_DELETION	ATM	D
1-06248	11	108139313	AC	A	FRAMESHIFT_DELETION	ATM	D
1-00668	11	108170565	G	A	STOP_GAINED	ATM	R
1-02455	11	108202286	T	C	SPLICE_SITE_DONOR	ATM	R
1-05019	11	108206605	C	T	STOP_GAINED	ATM	D
1-10274	11	118368773	CA	C	FRAME_SHIFT	KMT2A	R
S51A4OL7	11	119142446	C	T	STOP_GAINED&SPLICE_REGION_V/	CBL	R
1-04880	11	119170425	CGA	C	FRAMESHIFT_DELETION	CBL	D
1-01632	11	120295065	A	G	SPLICE_SITE	ARHGEF12	R
1-01183	11	120349099	G	C	SPLICE_SITE	ARHGEF12	R
1-05181	11	120349099	G	A	SPLICE_SITE	ARHGEF12	R
1-02580	11	128781631	C	T	STOP_GAINED	KCNJ5	D
1-12917	11	128781976	CT	C	FRAMESHIFT_DELETION	KCNJ5	R
1-00071	12	461400	TA	T	FRAMESHIFT_DELETION	KDM5A	R
1-00826	12	6788632	CTG	C	FRAMESHIFT_DELETION	ZNF384	R
1-01237	12	7055898	CG	C	FRAMESHIFT_VARIANT	PTPN6	R
1-13581	12	7055903	G	C	SPLICE_DONOR_VARIANT&INTRON	PTPN6	R
1-03148	12	7061234	G	T	STOPGAIN	PTPN6	D
1-11325	12	7067153	TG	T	FRAMESHIFT_VARIANT	PTPN6	R
1-15124	12	22778152	CG	C	FRAMESHIFT_VARIANT	ETNK1	R
1-01528	12	27824480	G	T	SPLICE_SITE	PPFIBP1	R
S86S9595	12	27840401	CTT	C	FRAMESHIFT_VARIANT	PPFIBP1	D
S86S9595	12	27840406	A	AGG	FRAMESHIFT_VARIANT	PPFIBP1	D
1-02566	12	49420016	CTT	C	FRAME_SHIFT	KMT2D	R
GT04016063	12	49420510	ACATT	A	FRAME_SHIFT	KMT2D	R
1-00479	12	49424494	CAA	C	FRAME_SHIFT	KMT2D	R
1-08173	12	49424686	TGTTTCAGCA	T	STOP_GAINED	KMT2D	R
1-10799	12	49424741	G	A	STOP_GAINED	KMT2D	D
1-05572	12	49425896	G	A	STOP_GAINED	KMT2D	D

© 2020 Morton SU et al. JAMA Cardiology.

Blinded.ID	Chromosome	Position	Reference	Alternate	Variant Effect	Gene	Cohort*
1-07473	12	49427848	A	T	SPLICE_SITE_DONOR	KMT2D	D
1-00054	12	49435286	C	CA	FRAMESHIFT_VARIANT	KMT2D	D
1-02838	12	49436629	G	A	STOPGAIN	KMT2D	D
1-07570	12	49437701	G	A	STOP_GAINED	KMT2D	D
1-00596	12	49438004	CA	C	FRAME_SHIFT	KMT2D	D
GT04008173	12	49446763	A	T	STOP_GAINED	KMT2D	R
1-00336	12	49447257	AC	A	SPLICE_DONOR_VARIANT&INTRON	KMT2D	D
GT04010993	12	49447337	G	GC	FRAME_SHIFT	KMT2D	D
1-12480	12	49448767	G	C	STOP_GAINED	KMT2D	R
1-06174	12	50025278	TCCTGCCC	T	FRAMESHIFT_DELETION	PRPF40B	R
1-02173	12	51208085	A	AT	FRAMESHIFT_VARIANT	ATF1	R
1-04877	12	56478777	A	C	SPLICE_ACCEPTOR_VARIANT&INTR	ERBB3	D
1-13251	12	56490996	G	A	STOP_GAINED	ERBB3	D
1-09032	12	56495753	AG	A	FRAMESHIFT_DELETION	ERBB3	D
1-10800	12	57115067	GA	G	FRAMESHIFT_DELETION	NACA	D
SQ5R3D9I	12	57498939	AG	A	FRAMESHIFT_VARIANT	STAT6	D
1-01021	12	57860074	TG	T	FRAMESHIFT_DELETION	GLI1	R
1-02634	12	57863470	T	A	STOPGAIN	GLI1	D
GT04011011	12	57910728	T	TG	FRAMESHIFT_INSERTION	DDIT3	R
1-03630	12	57910957	AT	A	FRAMESHIFT_DELETION	DDIT3	D
1-07129	12	57910962	TC	T	FRAMESHIFT_DELETION	DDIT3	R
1-03680	12	59266489	TG	T	FRAMESHIFT_VARIANT	LRIG3	R
1-05147	12	59268211	C	T	SPLICE_SITE	LRIG3	R
1-06922	12	59271222	GT	G	FRAMESHIFT_DELETION	LRIG3	R
1-02568	12	65449810	C	T	SPLICE_SITE	WIF1	R
GT04011011	12	65462542	A	G	SPLICE_SITE	WIF1	R
1-03667	12	65514297	AT	A	FRAMESHIFT_VARIANT	WIF1	D
SHGI83M4	12	71003594	T	C	START_LOST	PTPRB	D
1-10165	12	95922765	GC	G	FRAMESHIFT_VARIANT	USP44	D
1-00750	12	95926925	G	A	STOPGAIN	USP44	D
1-03173	12	95927441	C	CT	FRAMESHIFT_INSERTION	USP44	D
1-06600	12	95927447	C	A	STOPGAIN	USP44	R
1-13202	12	115112349	G	GCA	FRAMESHIFT_INSERTION	TBX3	R
1-03484	12	115112505	AC	A	FRAMESHIFT_DELETION	TBX3	D
1-07174	12	122763687	GA	G	FRAMESHIFT_DELETION	CLIP1	D
1-04910	12	124811954	C	T	SPLICE_SITE	NCOR2	R
1-10653	12	124887096	T	TGCTGC	FRAMESHIFT_VARIANT	NCOR2	D
1-03665	12	133210945	CCT	C	FRAMESHIFT_DELETION	POLE	R
1-00301	12	133225553	G	A	STOP_GAINED	POLE	R
SL865M57	12	133249752	CG	C	FRAMESHIFT_VARIANT	POLE	D
S85RV7M4	13	21557362	C	A	SPLICE_DONOR_VARIANT&INTRON	LATS2	R
1-01221	13	32905148	AAG	A	FRAMESHIFT_DELETION	BRCA2	D
1-01631	13	32912212	GT	G	FRAMESHIFT_VARIANT	BRCA2	R
1-06797	13	32912375	C	T	STOPGAIN	BRCA2	R
1-06167	13	32912414	G	T	STOPGAIN	BRCA2	R
1-00174	13	32912767	T	TA	FRAMESHIFT_INSERTION	BRCA2	D
1-05254	13	32913118	GA	G	FRAMESHIFT_DELETION	BRCA2	R
S9232D67	13	32913182	G	GC	FRAMESHIFT_VARIANT	BRCA2	D
1-10851	13	32931913	AAATT	A	FRAMESHIFT_VARIANT	BRCA2	D
1-04879	13	32937315	G	C	SPLICE_SITE_ACCEPTOR	BRCA2	R
1-07590	13	46718575	A	G	SPLICE_SITE	LCP1	R
1-03344	13	46721148	G	A	STOP_GAINED	LCP1	R
1-07126	13	48916734	G	A	SPLICE_SITE	RB1	R
1-03394	13	92345488	C	CT	FRAMESHIFT_INSERTION	GPC5	R

© 2020 Morton SU et al. JAMA Cardiology.

Blinded.ID	Chromosome	Position	Reference	Alternate	Variant Effect	Gene	Cohort*
1-02174	13	92380840	TC	T	FRAMESHIFT_DELETION	GPC5	D
1-02153	14	35230973	TC	T	FRAMESHIFT_DELETION	BAZ1A	D
1-12803	14	51190346	A	C	STOP_GAINED	NIN	R
1-12803	14	51190346	A	C	STOP_GAINED	NIN	R
1-02893	14	51208386	CAG	C	FRAMESHIFT_DELETION	NIN	R
1-04800	14	51211043	A	AT	FRAMESHIFT_INSERTION	NIN	D
1-04246	14	51223237	TCA	T	FRAMESHIFT_DELETION	NIN	R
1-05293	14	51224568	C	CA	FRAMESHIFT_VARIANT	NIN	D
1-03554	14	51225305	C	CTA	FRAMESHIFT_VARIANT	NIN	R
1-06465	14	51237706	G	A	STOP_GAINED	NIN	R
1-07299	14	56096685	CAG	C	FRAMESHIFT_DELETION	KTN1	R
1-12220	14	56106626	TA	T	SPLICE_ACCEPTOR_VARIANT&INTR	KTN1	R
1-05055	14	56114766	TAA	T	FRAMESHIFT_DELETION	KTN1	R
1-06290	14	56138580	CAA	C	FRAMESHIFT_DELETION	KTN1	R
S767M363	14	68331700	TTTTTTTTTTT	T	SPLICE_ACCEPTOR_VARIANT&SPLI	RAD51B	D
1-08920	14	68331714	T	TTTTTTTTTTTTTTTTT	SPLICE_ACCEPTOR_VARIANT&INTR	RAD51B	D
1-06988	14	68352706	G	A	SPLICE_SITE	RAD51B	D
S8BFH5D3	14	81422043	C	CTGCA	FRAMESHIFT_VARIANT	TSHR	D
1-00658	14	81606166	ACC	A	FRAMESHIFT_DELETION	TSHR	D
1-03565	14	92472214	CTGAT	C	FRAMESHIFT_DELETION	TRIP11	D
1-01564	14	92472720	G	A	STOPGAIN	TRIP11	D
1-01687	14	92491765	CT	C	SPLICE_SITE	TRIP11	R
1-12767	14	93286149	G	T	STOPGAIN	GOLGA5	D
SQ4CNIBB	14	95562261	A	ACC	FRAMESHIFT_VARIANT	DICER1	D
1-03656	14	99640499	C	A	STOPGAIN	BCL11B	D
1-05659	14	102551272	CTTCT	C	FRAMESHIFT_DELETION	HSP90AA1	R
1-09347	14	102568259	GC	G	FRAMESHIFT_DELETION	HSP90AA1	D
1-12475	14	105242036	C	CTGAGTTG	FRAMESHIFT_VARIANT	AKT1	D
SQ5M9176	15	34635847	C	A	STOP_GAINED	NUTM1	D
SP3NE597	15	34647220	A	G	SPLICE_ACCEPTOR_VARIANT&INTR	NUTM1	R
1-02944	15	34647917	C	T	STOPGAIN	NUTM1	D
1-02789	15	34649147	A	AGG	FRAMESHIFT_INSERTION	NUTM1	R
1-06524	15	34649516	C	T	STOPGAIN	NUTM1	R
1-08425	15	40502360	C	G	STOPGAIN	BUB1B	D
SRFX86GG	15	40505560	AC	A	FRAMESHIFT_VARIANT	BUB1B	D
1-03195	15	40895165	G	GT	SPLICE_SITE_DONOR	CASC5	R
1-00096	15	40914902	GA	G	FRAMESHIFT_DELETION	CASC5	D
1-15150	15	40947176	G	A	SPLICE_DONOR_VARIANT&INTRON	CASC5	R
S38BLVQJ	15	45007918	T	A	STOP_GAINED	B2M	D
GT04013752	15	50782004	C	T	STOPGAIN	USP8	D
1-07011	15	50789398	T	TA	FRAME_SHIFT	USP8	R
1-05866	15	52646177	G	T	STOPGAIN	MYO5A	R
1-05753	15	57364875	T	A	STOP_GAINED	TCF12	R
1-03077	15	57523399	GTTATCCA	G	FRAMESHIFT_DELETION	TCF12	D
1-02940	15	57545466	C	T	STOP_GAINED	TCF12	R
1-14322	15	57554311	ACT	A	FRAMESHIFT_VARIANT	TCF12	D
1-06058	15	57555309	G	A	SPLICE_SITE	TCF12	R
GT04013633	15	67430432	TG	T	FRAMESHIFT_DELETION	SMAD3	R
1-02775	15	90631821	G	A	STOPGAIN	IDH2	D
1-00839	15	91304231	TAGAA	T	FRAMESHIFT_DELETION	BLM	R
1-06168	15	91341513	CAT	C	FRAMESHIFT_DELETION	BLM	D
1-05805	15	91346866	CTT	C	FRAMESHIFT_DELETION	BLM	R
S389MD2B	15	91354587	C	T	STOP_GAINED	BLM	D
1-06339	15	91434410	C	T	STOPGAIN	FES	D

© 2020 Morton SU et al. JAMA Cardiology.

Blinded.ID	Chromosome	Position	Reference	Alternate	Variant Effect	Gene	Cohort*
1-06006	15	91434410	C	T	STOPGAIN	FES	R
1-00277	16	2131781	C	CT	FRAMESHIFT_INSERTION	TSC2	R
1-06909	16	2138446	G	C	SPLICE_SITE	TSC2	D
1-03358	16	2226387	T	G	SPLICE_SITE	TRAF7	R
1-07406	16	3820792	G	A	STOP_GAINED	CREBBP	R
1-07406	16	3820792	G	A	STOP_GAINED	CREBBP	R
1-02084	16	9934865	G	C	STOP_GAINED	GRIN2A	R
1-12080	16	11410729	G	GC	FRAMESHIFT_VARIANT	RMI2	R
SC32I193	16	11439366	CG	C	FRAMESHIFT_VARIANT	RMI2	R
1-02767	16	14041767	C	T	STOPGAIN	ERCC4	D
1-03325	16	14042136	A	T	STOPGAIN	ERCC4	R
1-04585	16	15797979	G	A	STOPGAIN	MYH11	R
1-17622	16	15831479	T	C	SPLICE_ACCEPTOR_VARIANT&INTR	MYH11	D
GT04010993	16	23619211	G	C	STOPGAIN	PALB2	D
1-10029	16	23634364	CTT	C	FRAMESHIFT_VARIANT	PALB2	R
1-10274	16	23637587	C	T	STOPGAIN	PALB2	R
1-03835	16	23637594	C	T	STOPGAIN	PALB2	R
1-01407	16	23646674	AC	A	FRAMESHIFT_DELETION	PALB2	R
1-00117	16	23649420	C	A	STOPGAIN	PALB2	D
1-09426	16	50788336	G	A	SPLICE_SITE	CYLD	R
1-02496	16	50826561	AT	A	FRAMESHIFT_DELETION	CYLD	R
1-05660	16	56966172	G	T	STOPGAIN	HERPUD1	D
1-02084	16	72984604	G	A	STOP_GAINED	ZFHX3	R
1-02084	16	72984604	G	A	STOP_GAINED	ZFHX3	R
SU68YRYV	16	74671821	G	GA	FRAMESHIFT_VARIANT	RFWD3	R
1-07218	16	89805692	AG	A	FRAMESHIFT_VARIANT	FANCA	D
1-00077	16	89806403	ACT	A	FRAMESHIFT_VARIANT&SPLICE_RE	FANCA	R
S22VYLQ4	16	89809210	CCT	C	FRAMESHIFT_VARIANT	FANCA	D
1-06195	16	89809301	C	T	STOPGAIN	FANCA	R
1-02498	16	89811472	C	T	STOPGAIN	FANCA	R
1-07361	16	89833613	CAG	C	FRAMESHIFT_VARIANT	FANCA	R
1-00702	16	89836246	T	A	STOPGAIN	FANCA	R
1-01966	16	89846347	G	A	STOPGAIN	FANCA	R
1-14708	16	89869741	G	A	STOP_GAINED	FANCA	D
1-00620	17	5253892	C	T	STOPGAIN	RABEP1	R
1-04097	17	5283735	TAAAG	T	FRAMESHIFT_DELETION	RABEP1	R
1-06021	17	7578263	G	A	STOP_GAINED	TP53	D
1-06197	17	7578555	C	CT	SPLICE_ACCEPTOR_VARIANT&INTR	TP53	R
1-00559	17	8053096	A	ACG	FRAMESHIFT_INSERTION	PER1	D
1-01905	17	9873017	G	A	STOPGAIN	GAS7	D
1-09185	17	9963008	C	T	SPLICE_DONOR_VARIANT&INTRON	GAS7	D
1-09185	17	9963008	C	T	SPLICE_DONOR_VARIANT&INTRON	GAS7	D
1-00018	17	20200305	G	A	STOPGAIN	SPECC1	D
1-01124	17	29509596	G	A	STOPGAIN	NF1	D
1-00941	17	29663350	G	T	SPLICE_SITE	NF1	D
1-00749	17	34172011	C	T	STOPGAIN	TAF15	D
1-08730	17	37868702	G	A	SPLICE_SITE	ERBB2	R
1-00064	17	37873705	C	T	STOPGAIN	ERBB2	D
1-06413	17	41209095	G	A	STOPGAIN	BRCA1	R
1-03963	17	41243869	G	A	STOPGAIN	BRCA1	D
1-13184	17	41246748	GAA	G	FRAMESHIFT_VARIANT	BRCA1	D
1-00748	17	41606535	G	A	STOPGAIN	ETV4	R
1-00544	17	41610569	G	T	STOPGAIN	ETV4	R
1-02787	17	41622925	C	A	SPLICE_DONOR_VARIANT&INTRON	ETV4	D

© 2020 Morton SU et al. JAMA Cardiology.

Blinded.ID	Chromosome	Position	Reference	Alternate	Variant Effect	Gene	Cohort*
1-05425	17	47700239	C	CTGGTCAGAT	SPLICE_ACCEPTOR_VARIANT&INTR	SPOP	D
1-04112	17	48267714	CG	C	FRAMESHIFT_DELETION	COL1A1	R
1-02038	17	58740836	C	T	STOPGAIN	PPM1D	D
1-01660	17	59878698	A	ATATG	FRAMESHIFT_INSERTION	BRIP1	R
1-12803	17	62502379	T	C	SPLICE_ACCEPTOR_VARIANT&INTR	DDX5	R
1-13288	17	62502379	T	C	SPLICE_ACCEPTOR_VARIANT&INTR	DDX5	R
1-00148	17	63530130	AG	A	FRAMESHIFT_DELETION	AXIN2	D
1-04881	17	63554325	G	C	STOPGAIN	AXIN2	D
1-02795	17	63554453	G	A	STOPGAIN	AXIN2	D
1-05570	17	75471643	CTGCCGCAGG	C	FRAMESHIFT_DELETION	9-Sep	R
1-02231	17	76989789	AT	A	FRAMESHIFT_DELETION	CANT1	R
1-00788	17	76991237	C	T	STOPGAIN	CANT1	D
S3LE5711	17	76991292	C	A	STOP_GAINED	CANT1	R
1-05752	17	76993317	T	A	STOP_GAINED	CANT1	R
1-02987	17	78269356	G	A	SPLICE_SITE	RNF213	R
1-03879	17	78286962	A	AT	FRAMESHIFT_INSERTION	RNF213	R
1-06213	17	78311709	T	G	STOPGAIN	RNF213	D
1-03105	17	78313267	C	A	STOPGAIN	RNF213	D
SSDIX83M	17	78318837	CT	C	FRAMESHIFT_VARIANT	RNF213	R
1-02903	17	78333985	C	T	STOPGAIN	RNF213	R
1-03537	17	78358944	A	T	STOPGAIN	RNF213	D
1-03809	17	79969418	G	T	SPLICE_SITE_ACCEPTOR	ASPSCR1	D
1-05371	17	79970106	GTC	G	FRAMESHIFT_DELETION	ASPSCR1	D
1-01817	18	22804351	TTGGGGCG	T	FRAME_SHIFT	ZNF521	D
1-04890	18	45368248	CT	C	FRAMESHIFT_DELETION	SMAD2	D
1-02020	18	45374845	C	T	SPLICE_SITE_DONOR	SMAD2	D
1-04276	18	61002545	G	T	STOPGAIN	KDSR	D
1-00730	18	61022533	C	T	SPLICE_SITE	KDSR	D
1-11091	19	4102432	TG	T	FRAMESHIFT_VARIANT	MAP2K2	R
1-01733	19	4362735	T	C	SPLICE_SITE	SH3GL1	D
1-16686	19	4363770	C	A	STOP_GAINED	SH3GL1	R
1-02692	19	7808112	C	T	STOPGAIN	CD209	D
SC72XCU6	19	7809980	T	G	SPLICE_ACCEPTOR_VARIANT&INTR	CD209	D
1-11578	19	8961981	CT	C	FRAMESHIFT_VARIANT	MUC16	D
1-06757	19	8976433	C	A	SPLICE_SITE	MUC16	D
1-07644	19	8996414	T	A	STOPGAIN	MUC16	R
S3Q536JN	19	8998700	G	T	STOP_GAINED&SPLICE_REGION_V/	MUC16	R
1-07513	19	8999023	A	C	SPLICE_SITE	MUC16	R
1-02327	19	8999061	C	A	SPLICE_SITE	MUC16	D
1-06369	19	9003565	C	A	SPLICE_DONOR_VARIANT&INTRON	MUC16	D
1-03383	19	9024132	A	T	SPLICE_SITE	MUC16	R
SFZ94QV5	19	9045572	G	C	STOP_GAINED	MUC16	D
1-05752	19	9045572	G	C	STOP_GAINED	MUC16	R
1-01301	19	9046084	TG	T	FRAMESHIFT_DELETION	MUC16	D
1-00893	19	9054234	C	A	SPLICE_SITE	MUC16	R
1-03720	19	9066613	ACT	A	FRAMESHIFT_VARIANT	MUC16	R
1-11005	19	9077405	C	T	STOP_GAINED	MUC16	D
1-07168	19	9082584	TG	T	FRAMESHIFT_DELETION	MUC16	D
1-03480	19	9082673	A	AT	FRAMESHIFT_INSERTION	MUC16	R
1-06809	19	9087723	G	T	STOPGAIN	MUC16	D
1-02723	19	9090041	C	A	STOPGAIN	MUC16	R
S4S9NTZ9	19	10600480	G	A	STOP_GAINED	KEAP1	D
1-02084	19	14217633	A	G	START_LOST	PRKACA	R
1-02437	19	14626809	GA	G	FRAME_SHIFT	DNAJB1	D

© 2020 Morton SU et al. JAMA Cardiology.

Blinded.ID	Chromosome	Position	Reference	Alternate	Variant Effect	Gene	Cohort*
1-10358	19	15364543	CTG	C	FRAMESHIFT_VARIANT	BRD4	R
1-05286	19	17937718	AC	A	FRAMESHIFT_DELETION	JAK3	R
1-02452	19	21688577	G	T	SPLICE_SITE	ZNF429	D
1-18335	19	21720417	CCTCAAGACT	C	FRAMESHIFT_VARIANT	ZNF429	D
1-06907	19	21720550	C	A	STOPGAIN	ZNF429	R
S134MA7T	19	30313351	AG	A	FRAMESHIFT_VARIANT&SPLICE_RE	CCNE1	D
1-01684	19	33439223	G	A	STOPGAIN	CEP89	R
1-05272	19	45295751	T	TGCTGCCTG	FRAMESHIFT_INSERTION	CBLC	R
1-05488	19	45860755	G	A	STOP_GAINED	ERCC2	D
1-07513	19	49464240	GC	G	FRAMESHIFT_DELETION	BAX	R
1-04478	19	54081177	C	T	STOPGAIN	ZNF331	R
1-00347	20	20020429	G	A	STOPGAIN	CRNKL1	R
1-04603	20	31024758	C	T	STOP_GAINED	ASXL1	D
1-03873	20	39794189	G	T	STOPGAIN	PLCG1	D
1-03656	20	39798866	G	A	STOPGAIN	PLCG1	D
S8J8C4X9	20	39801459	T	TC	FRAMESHIFT_VARIANT	PLCG1	R
1-07518	20	40828039	T	C	SPLICE_ACCEPTOR_VARIANT&INTR	PTPRT	R
SGO97745	20	43959206	T	C	SPLICE_ACCEPTOR_VARIANT&INTR	SDC4	R
1-04961	20	57415222	TGCCC	T	FRAMESHIFT_DELETION	GNAS	R
1-04355	20	57429002	CT	C	FRAMESHIFT_DELETION	GNAS	R
1-06105	20	60733726	A	G	SPLICE_SITE	SS18L1	D
SY5BRA48	20	60737839	GCCTAACACG	G	FRAMESHIFT_VARIANT	SS18L1	D
1-06541	20	62164943	T	A	STOPGAIN	PTK6	R
1-04606	20	62168463	C	A	STOPGAIN	PTK6	D
1-03095	21	39772504	GT	G	FRAMESHIFT_DELETION	ERG	D
1-00561	22	19203682	TA	T	FRAMESHIFT_DELETION	CLTCL1	D
SBX89YO5	22	19213907	C	T	SPLICE_ACCEPTOR_VARIANT&INTR	CLTCL1	D
1-03470	22	19223381	TTTAG	T	FRAMESHIFT_DELETION	CLTCL1	D
1-03905	22	19263251	G	A	STOPGAIN	CLTCL1	R
S17ZO6BY	22	21340187	G	C	SPLICE_DONOR_VARIANT&INTRON	LZTR1	R
GT04013872	22	21342297	A	G	SPLICE_SITE	LZTR1	R
1-06553	22	21343972	G	T	SPLICE_SITE_DONOR	LZTR1	D
1-01700	22	21344770	CA	C	FRAMESHIFT_VARIANT	LZTR1	R
S742ZMBG	22	21346119	G	A	SPLICE_DONOR_VARIANT&INTRON	LZTR1	R
1-17063	22	21346119	G	A	SPLICE_DONOR_VARIANT&INTRON	LZTR1	R
SW4B94F9	22	21350066	C	G	STOP_GAINED	LZTR1	D
1-03590	22	21351012	C	A	STOPGAIN	LZTR1	D
1-15325	22	28194933	T	TGC	FRAMESHIFT_VARIANT	MN1	D
1-04465	22	28195640	G	GCT	FRAMESHIFT_INSERTION	MN1	R
1-05176	22	29095881	CG	C	FRAMESHIFT_DELETION	CHEK2	R
SO9DBKP2	22	29446077	GCA	G	FRAMESHIFT_VARIANT	ZNRF3	D
1-04358	22	35478510	G	A	SPLICE_SITE_ACCEPTOR	ISX	D
1-00344	22	35481578	AAC	A	FRAMESHIFT_DELETION	ISX	D
1-02464	22	41564490	TC	T	FRAME_SHIFT	EP300	R
1-04567	22	41574502	C	T	STOPGAIN	EP300	D
1-06149	X	41203501	C	T	STOPGAIN	DDX3X	R
1-13106	X	44929551	C	A	STOP_GAINED	KDM6A	D
1-06087	X	48649565	C	T	STOP_GAINED	GATA1	D
1-02312	X	48887959	CT	C	FRAME_SHIFT	TFE3	D
1-01088	X	63412121	TG	T	FRAMESHIFT_DELETION	AMER1	D
1-01483	X	123184968	T	TTTTTA	SPLICE_ACCEPTOR_VARIANT&INTR	STAG2	R
1-09568	X	133527984	T	C	SPLICE_SITE_DONOR	PHF6	D
1-06072	X	153588421	G	A	STOPGAIN	FLNA	R

© 2020 Morton SU et al. JAMA Cardiology.

Blinded.ID	Chromosome	Position	Reference	Alternate	Variant Effect	Gene	Cohort*

*D=Discovery, R=Replication

© 2020 Morton SU et al. JAMA Cardiology.

eTable 5. Discovery Cohort Analysis

Gene List (Number)	CHD, 2222	Non-CHD, 3578	OR (95%CI)	Binomial p-value[1]
**All COSMIC (723)**	326	389	**1.41 (1.20-1.65)**	**2.20E-08**
**OMIM··· CHD (38)**	29	10	**4.72 (2.29-9.70)**	**2.55E-11**
**Regulatory (216)**	70	64	**1.79 (1.27-2.52)**	**7.35-06**
**Regulatory + OMIM[b] CHD (17)**	15	6	**4.05 (1.57-10.44)**	**8.62E-06**
**Regulatory - OMIM[b] CHD (199)**	55	58	1.54 (1.06-2.24)	1.79E-03
**Non-OMIM[b] CHD (685)**	302	379	**1.33 (1.13-1.56)**	**5.43E-06**
**Non-Regulatory (507)**	265	328	**1.34 (1.13-1.59)**	**8.47E-06**
**LoF Cancer Mechanism (227)**	121	151	1.31 (1.02-1.67)	3.26E-03
**-Recessive LoF (135)[c]**	79	100	1.28 (0.95-1.73)	2.03E-02
**-Dominant LoF (46)[c]**	31	22	**2.29 (1.32-3.96)**	**3.62E-05**
**Non-LoF Cancer Mechanism (496)**	213	251	**1.41 (1.16-1.70)**	**3.64E-06**

[a]Bonferroni significance p-value threshold: 10 gene lists x 3 comparisons, 1.67E-03. Bolded if also significant in both sub-analyses
[b]OMIM CHD genes with dominant patterns of transmission.
[c]5 genes have both dominant and recessive cancer variants, 51 are not characterized

© 2020 Morton SU et al. JAMA Cardiology.

eTable 6. Replication Cohort Analysis

Gene List (Number)	CHD, 2221	Non-CHD, 3578	OR (95%CI)	Binomial p-value[1]
**All COSMIC (723)**	316	710	**1.29 (1.12-1.49)**	**2.68E-05**
**OMIM··· CHD (38)**	39	8	**13.90 (6.49-29.79)**	**<2.2E-16**
**Regulatory (216)**	73	102	**2.04 (1.51-2.77)**	**4.37E-08**
**Regulatory + OMIM[b] CHD (17)**	25	3	**23.63 (7.13-78.34)**	**<2.2E-16**
**Regulatory - OMIM[b] CHD (199)**	48	99	**2.17 (1.53-3.07)**	**1.57E-06**
**Non-OMIM[b] CHD (685)**	283	703	1.15 (0.99-1.33)	1.75E-02
**Non-Regulatory (507)**	251	614	1.17 (1.00-1.36)	1.34E-02
**LoF Cancer Mechanism (227)**	119	225	**1.51 (1.20-1.90)**	**2.15E-05**
**-Recessive LoF (135)[c]**	79	174	1.28 (0.98-1.68)	1.98E-02
**-Dominant LoF (46)[c]**	22	28	**2.22 (1.27-3.88)**	**6.63E-04**
**Non-LoF Cancer Mechanism (496)**	209	500	1.19 (1.00-1.41)	2.03E-02

[a]Bonferroni significance p-value threshold: 10 gene lists x 3 comparisons, 1.67E-03. Bolded if also significant in both sub-analyses
[b]OMIM CHD genes with dominant patterns of transmission.
[c]5 genes have both dominant and recessive cancer variants, 51 are not characterized

© 2020 Morton SU et al. JAMA Cardiology.

eTable 7. Cohort Subset Analysis

CHD Type	CR Variant, 551	No-CR Variant, 3277	OR (95%CI)	Binomial p-value*
Endocardial Cushion Defect	51	213	1.47	0.02
Abnormal Looping	18	179	0.58	0.03
Double inlet left ventricle	7	68	0.61	0.21
Ebstein anomaly	6	54	0.66	0.33
Conotruncaldefect	192	1117	1.03	0.73
Right outflow tract abnormality	19	104	1.09	0.74
Left outflo abnormality	157	948	0.98	0.83
Tricuspid atresia	12	74	0.96	0.91
Heterotaxy	28	164	1.02	0.94
Isolated atrial septal defect	11	67	0.98	0.94
Total anomalous pulmonary venous return	15	90	0.99	0.97
Isolated ventricular septal defect	7	42	0.99	0.98

[a]Bonferroni significant p-value threshold 2.08 E-03

© 2020 Morton SU et al. JAMA Cardiology.

eTable 8. Per-gene count of all loss-of-function variants

Gene	Total CHD	Total Control	pLI	Heart Expression Rank	Binomial p
NOTCH1	13	0	1.00	88	2.63E-07
RUNX1T1	1	0	1.00	84	2.89E-07
DDX3X	1	0	1.00	97	2.89E-07
KMT2D	15	1	1.00	97	2.86E-04
MAML2	1	0	1.00	67	5.81E-04
IL6ST	1	0	1.00	91	1.90E-03
FLT4	15	1	1.00	74	2.95E-03
GRIN2A	1	0	1.00	14	4.71E-03
KDR	7	0	0.98	87	9.45E-03
ELN	8	1	0.00	80	9.45E-03
EIF4A2	1	0	1.00	96	1.31E-02
CTNND1	1	0	1.00	99	1.31E-02
NSD1	5	1	1.00	95	3.03E-02
PTPN6	4	0	1.00	36	3.03E-02
CTNNB1	4	0	1.00	99	3.03E-02
TAL1	5	0	0.82	55	3.03E-02
MUC1	6	1	0.73	22	3.03E-02
NUTM1	5	1	0.01	13	3.03E-02
POLQ	13	7	0.00	42	3.03E-02
ASXL2	1	0	0.99	76	3.40E-02
SPOP	1	0	0.99	90	3.40E-02
SEPT9	1	0	0.89	92	3.94E-02
WWTR1	1	0	0.92	84	6.18E-02
BCL11B	1	0	0.93	49	6.76E-02
IRF4	1	0	0.92	21	8.02E-02
MSH2	1	0	0.87	76	9.23E-02
ESR1	1	0	0.99	29	9.29E-02
TFE3	1	0	0.97	74	9.29E-02
PHF6	1	0	0.97	57	9.29E-02
LEF1	1	0	0.97	35	9.29E-02
ERG	1	0	0.97	61	9.29E-02
PRKACA	1	0	0.96	98	9.29E-02
NPM1	1	0	0.96	98	9.29E-02
PSIP1	1	0	0.95	92	9.29E-02
IGF2BP2	1	0	0.94	85	9.29E-02
ZNF384	1	0	0.94	79	9.29E-02
CREBBP	2	0	1.00	90	9.72E-02
KMT2C	2	0	1.00	80	9.72E-02
TSC2	2	0	1.00	93	9.72E-02
CAMTA1	2	0	1.00	91	9.72E-02
KAT6B	2	0	1.00	82	9.72E-02
NCOR2	2	0	1.00	92	9.72E-02
NUMA1	2	0	1.00	95	9.72E-02

© 2020 Morton SU et al. JAMA Cardiology.

Gene	Total CHD	Total Control	pLI	Heart Expression Rank	Binomial p
ARHGEF12	3	0	1.00	91	9.72E-02
USP8	2	0	1.00	84	9.72E-02
MLLT10	3	0	1.00	85	9.72E-02
TET1	2	0	1.00	68	9.72E-02
RAD21	2	0	1.00	96	9.72E-02
ERBB2	2	0	1.00	94	9.72E-02
EPAS1	2	0	1.00	86	9.72E-02
DDX5	2	0	1.00	99	9.72E-02
ETV1	2	0	1.00	78	9.72E-02
DROSHA	2	0	1.00	91	9.72E-02
RPL5	3	0	0.99	98	9.72E-02
CCNC	2	0	0.99	57	9.72E-02
SMAD2	2	0	0.99	75	9.72E-02
RABEP1	2	0	0.98	74	9.72E-02
PRDM1	2	0	0.98	30	9.72E-02
CUL3	2	0	0.97	83	9.72E-02
TCF12	5	2	0.97	89	9.72E-02
TBX3	2	0	0.95	44	9.72E-02
NRG1	2	0	0.95	33	9.72E-02
TP53	2	0	0.91	89	9.72E-02
PAX3	2	0	0.90	0	9.72E-02
BIRC3	3	0	0.89	41	9.72E-02
SS18L1	2	0	0.81	49	9.72E-02
KIF5B	2	0	0.76	97	9.72E-02
MLH1	2	0	0.74	61	9.72E-02
DDIT3	3	0	0.52	55	9.72E-02
GAS7	3	0	0.03	56	9.72E-02
CASP8	3	0	0.00	51	9.72E-02
TNC	5	2	0.00	66	9.72E-02
PTPRK	3	3	0.98	90	1.26E-01
POT1	1	0	0.52	49	1.38E-01
MAP2K2	1	0	0.86	85	1.42E-01
B2M	1	0	0.86	93	1.42E-01
GATA1	1	0	0.84	22	1.42E-01
PLAG1	1	0	0.83	40	1.42E-01
CNTNAP2	4	4	0.00	35	1.77E-01
RHOA	1	0	0.80	97	1.79E-01
FGFR1OP	2	2	0.71	69	1.79E-01
RSPO2	1	0	0.69	29	1.79E-01
NFE2L2	1	0	0.69	78	1.79E-01
ZNF331	1	0	0.68	0	1.79E-01
ATF1	1	0	0.66	58	1.79E-01
ABI1	1	0	0.63	83	1.79E-01
NIN	8	9	0.00	70	2.16E-01

© 2020 Morton SU et al. JAMA Cardiology.

Gene	Total CHD	Total Control	pLI	Heart Expression Rank	Binomial p
TRAF7	1	0	0.46	89	2.31E-01
HIST1H4I	1	0	0.45	34	2.31E-01
IDH2	1	0	0.38	100	2.31E-01
DNAJB1	1	0	0.37	81	2.31E-01
GOLGA5	1	0	0.36	70	2.31E-01
CCNE1	1	0	0.27	54	2.31E-01
KEAP1	1	0	0.25	82	2.31E-01
STAT6	1	0	0.10	77	2.31E-01
TGFBR2	1	0	0.04	86	2.31E-01
VHL	1	0	0.03	57	2.31E-01
GOPC	1	0	0.02	63	2.31E-01
RAD17	1	0	0.01	65	2.31E-01
FANCF	1	0	0.00	37	2.31E-01
MYC	1	0	0.00	69	2.31E-01
CDH17	1	0	0.00	0	2.31E-01
MUTYH	1	0	0.00	41	2.31E-01
PLCG1	3	3	0.76	83	2.78E-01
LPP	3	3	0.58	91	2.78E-01
EPS15	3	3	0.24	76	2.78E-01
ETV4	3	3	0.00	43	2.78E-01
LIFR	3	3	0.00	77	2.78E-01
FANCA	9	11	0.00	57	2.78E-01
MYH11	2	2	1.00	36	3.08E-01
EP300	2	1	1.00	88	3.12E-01
MTOR	2	1	1.00	80	3.12E-01
KMT2A	1	0	1.00	86	3.12E-01
TRRAP	1	0	1.00	92	3.12E-01
COL1A1	1	0	1.00	99	3.12E-01
COL3A1	2	1	1.00	99	3.12E-01
CSMD3	8	6	1.00	15	3.12E-01
NOTCH2	3	1	1.00	85	3.12E-01
FLNA	1	0	1.00	99	3.12E-01
PTPRD	1	0	1.00	83	3.12E-01
SF3B1	1	0	1.00	95	3.12E-01
STAG1	1	0	1.00	78	3.12E-01
RB1	1	0	1.00	49	3.12E-01
FAT3	3	1	1.00	48	3.12E-01
STAG2	1	0	1.00	82	3.12E-01
WHSC1	1	0	1.00	89	3.12E-01
PTPRT	1	0	1.00	22	3.12E-01
PTCH1	1	0	1.00	71	3.12E-01
MLLT4	3	2	1.00	92	3.12E-01
KDM5A	1	0	1.00	86	3.12E-01
BRD4	1	0	1.00	95	3.12E-01

© 2020 Morton SU et al. JAMA Cardiology.

Gene	Total CHD	Total Control	pLI	Heart Expression Rank	Binomial p
KDM6A	1	0	1.00	71	3.12E-01
ZNF521	1	0	1.00	79	3.12E-01
FBXW7	1	0	1.00	61	3.12E-01
CDC73	1	0	1.00	52	3.12E-01
ARID1B	1	0	1.00	83	3.12E-01
FOXP1	1	0	1.00	94	3.12E-01
GNAS	2	1	1.00	100	3.12E-01
EXT1	1	0	1.00	81	3.12E-01
CYLD	2	1	0.99	59	3.12E-01
NUP214	3	2	0.99	78	3.12E-01
PTEN	3	1	0.98	78	3.12E-01
LCP1	2	1	0.97	75	3.12E-01
CBLB	3	1	0.91	72	3.12E-01
TFRC	3	1	0.78	82	3.12E-01
LMO2	2	1	0.75	49	3.12E-01
FIP1L1	2	1	0.60	85	3.12E-01
ALK	4	3	0.52	28	3.12E-01
AFF1	2	1	0.48	81	3.12E-01
KDSR	2	1	0.47	56	3.12E-01
LRIG3	3	1	0.43	31	3.12E-01
VTI1A	2	1	0.41	54	3.12E-01
KCNJ5	2	0	0.31	86	3.12E-01
AXIN2	3	1	0.12	50	3.12E-01
ACSL6	3	1	0.09	26	3.12E-01
SH3GL1	2	0	0.06	82	3.12E-01
NDRG1	3	2	0.05	68	3.12E-01
RSPO3	2	0	0.04	37	3.12E-01
CDKN1A	2	1	0.03	58	3.12E-01
RMI2	2	0	0.02	47	3.12E-01
CASC5	3	2	0.02	50	3.12E-01
ABL2	4	3	0.01	67	3.12E-01
NCKIPSD	4	2	0.01	44	3.12E-01
CBL	2	1	0.01	34	3.12E-01
DDB2	2	1	0.01	51	3.12E-01
FANCE	2	1	0.00	51	3.12E-01
CANT1	4	3	0.00	71	3.12E-01
TEC	2	0	0.00	60	3.12E-01
IDH1	2	0	0.00	74	3.12E-01
ISX	2	0	0.00	18	3.12E-01
MN1	2	0	0.00	59	3.12E-01
WIF1	3	2	0.00	39	3.12E-01
GLI1	2	1	0.00	27	3.12E-01
EXT2	3	1	0.00	78	3.12E-01
RAD51B	3	1	0.00	39	3.12E-01

© 2020 Morton SU et al. JAMA Cardiology.

Gene	Total CHD	Total Control	pLI	Heart Expression Rank	Binomial p
PALB2	6	5	0.00	44	3.12E-01
PREX2	4	3	0.00	60	3.12E-01
PPFIBP1	3	2	0.00	88	3.12E-01
PMS2	2	0	0.00	52	3.12E-01
ZNF429	3	2	0.00	28	3.12E-01
MACC1	5	3	0.00	17	3.12E-01
LZTR1	8	7	0.00	84	3.12E-01
HSP90AA1	2	2	0.68	96	3.69E-01
EML4	2	2	0.39	78	3.69E-01
FH	2	2	0.15	95	3.69E-01
SDHAF2	2	2	0.00	70	3.69E-01
TSHR	2	2	0.00	14	3.69E-01
CSF3R	2	2	0.00	25	3.69E-01
FGFR4	3	4	0.00	26	3.69E-01
CD209	2	2	0.00	0	3.69E-01
FANCG	2	2	0.00	48	3.69E-01
CYP2C8	2	2	0.00	10	3.69E-01
BRCA2	9	12	0.00	55	3.69E-01
BAZ1A	1	1	1.00	65	3.80E-01
XPC	3	4	0.00	55	3.80E-01
PMS1	3	4	0.00	42	3.80E-01
DICER1	1	1	1.00	86	3.82E-01
ZFHX3	2	3	1.00	81	4.65E-01
NF1	2	3	1.00	82	4.65E-01
USP44	4	7	0.00	17	4.78E-01
RFWD3	1	3	0.53	74	4.96E-01
ITGAV	3	5	0.48	76	4.96E-01
SMO	2	3	0.06	80	4.96E-01
GPC5	2	3	0.00	16	4.96E-01
TSC1	1	1	1.00	74	5.26E-01
PDGFRA	1	1	1.00	70	5.26E-01
EGFR	1	1	1.00	34	5.26E-01
MAP3K1	1	1	1.00	71	5.26E-01
PRDM2	1	1	1.00	85	5.26E-01
MYO5A	1	1	0.99	70	5.26E-01
PRPF40B	1	1	0.99	79	5.26E-01
ELF3	1	1	0.99	14	5.26E-01
AKT1	1	1	0.98	98	5.26E-01
AMER1	1	1	0.95	65	5.26E-01
PTPRB	1	1	0.94	61	5.26E-01
SMAD3	1	1	0.87	81	5.26E-01
ETNK1	1	1	0.87	82	5.26E-01
TAF15	1	1	0.35	91	5.26E-01
BAX	1	1	0.17	84	5.26E-01

© 2020 Morton SU et al. JAMA Cardiology.

Gene	Total CHD	Total Control	pLI	Heart Expression Rank	Binomial p
SPECC1	1	1	0.11	59	5.26E-01
SDC4	1	1	0.01	74	5.26E-01
HRAS	1	1	0.01	63	5.26E-01
PPM1D	1	1	0.00	55	5.26E-01
CBLC	1	1	0.00	20	5.26E-01
BMP5	1	1	0.00	73	5.26E-01
MPL	1	1	0.00	27	5.26E-01
MGMT	1	1	0.00	43	5.26E-01
PTPN13	5	7	0.00	78	5.26E-01
FAT1	4	6	0.00	94	5.26E-01
TPR	1	2	1.00	95	5.43E-01
BLM	4	7	0.00	65	5.43E-01
AFF3	1	2	1.00	51	5.54E-01
PIK3CB	1	2	1.00	47	5.68E-01
CARD11	1	2	1.00	25	5.68E-01
MET	1	2	1.00	42	6.05E-01
JAK3	1	2	1.00	66	6.05E-01
ZNRF3	1	2	1.00	61	6.05E-01
EPHA7	1	2	0.99	85	6.15E-01
GRM3	1	2	0.98	0	6.15E-01
LRP1B	2	4	1.00	9	6.74E-01
CUX1	2	4	1.00	89	6.74E-01
ZEB1	1	2	0.98	92	6.74E-01
PER1	1	2	0.88	84	6.74E-01
LATS2	1	2	0.87	78	6.74E-01
BCL9	1	2	0.83	90	6.74E-01
HERPUD1	1	2	0.82	76	6.74E-01
RGS7	1	2	0.78	17	6.74E-01
ATR	2	5	0.70	54	6.74E-01
KTN1	4	8	0.64	95	6.74E-01
FBLN2	1	2	0.63	79	6.74E-01
CREB3L2	1	2	0.61	97	6.74E-01
MB21D2	1	2	0.39	53	6.74E-01
BUB1B	2	5	0.00	90	6.74E-01
PDE4DIP	4	8	0.00	96	6.74E-01
FES	2	4	0.00	49	6.74E-01
ERBB3	3	6	0.00	40	6.74E-01
IL7R	1	2	0.00	0	6.74E-01
FANCD2	1	2	0.00	50	6.74E-01
POLE	3	6	0.00	70	6.74E-01
APC	1	3	1.00	85	6.95E-01
CLIP1	1	3	0.97	81	6.95E-01
ERCC3	2	5	0.00	63	6.95E-01
NBN	2	5	0.00	65	6.95E-01

© 2020 Morton SU et al. JAMA Cardiology.

Gene	Total CHD	Total Control	pLI	Heart Expression Rank	Binomial p
PAX8	1	3	0.90	16	7.17E-01
MSH6	2	6	0.00	80	7.67E-01
PTK6	2	6	0.00	0	7.67E-01
ACVR1	1	4	0.96	63	7.74E-01
RALGDS	1	3	0.30	64	7.76E-01
DCTN1	1	3	0.29	94	7.76E-01
CD74	1	3	0.05	30	7.76E-01
NTRK1	1	3	0.00	11	7.76E-01
ITK	1	3	0.00	18	7.76E-01
MLF1	1	3	0.00	95	7.76E-01
TRIP11	3	8	0.00	49	7.76E-01
CHEK2	1	3	0.00	43	7.76E-01
WRN	4	9	0.00	47	7.76E-01
ASXL1	1	3	0.00	85	7.76E-01
CLTCL1	4	9	0.00	0	7.76E-01
RNF213	7	15	0.00	70	7.76E-01
DNMT3A	1	3	0.00	97	7.76E-01
STIL	1	4	0.20	52	8.22E-01
CRNKL1	1	4	0.88	58	8.24E-01
NACA	1	4	0.02	0	8.46E-01
ERCC4	2	7	0.00	51	8.46E-01
CNBD1	1	4	0.00	0	8.46E-01
ARHGEF10	3	9	0.00	75	8.46E-01
BRCA1	3	10	0.00	54	8.46E-01
BAP1	1	6	0.39	82	8.53E-01
MUC4	3	11	0.00	16	8.61E-01
BARD1	3	11	0.00	57	8.61E-01
CASP9	1	6	0.00	63	9.12E-01
KAT6A	1	9	1.00	89	9.27E-01
FANCC	1	6	0.00	44	9.27E-01
ATIC	1	6	0.00	80	9.27E-01
CNTRL	8	25	0.00	72	9.27E-01
ATM	7	25	0.00	55	9.32E-01
BRIP1	1	10	0.00	41	9.52E-01
RECQL4	3	17	0.00	43	9.72E-01
AKAP9	7	28	0.00	89	9.76E-01
ERCC2	1	13	0.00	62	9.84E-01
MUC16	18	57	0.00	37	9.95E-01
ROS1	5	44	0.00	0	1.00E+00
ECT2L	2	30	0.00	15	1.00E+00
PCM1	2	36	0.00	92	1.00E+00
ASPSCR1	2	49	0.00	82	1.00E+00
CEP89	1	41	0.00	67	1.00E+00
ANK1	0	1	1.00	92	1.00E+00

© 2020 Morton SU et al. JAMA Cardiology.

Gene	Total CHD	Total Control	pLI	Heart Expression Rank	Binomial p
BIRC6	0	2	1.00	90	1.00E+00
MYH9	0	1	1.00	98	1.00E+00
RANBP2	0	2	1.00	76	1.00E+00
NUP98	0	2	1.00	87	1.00E+00
FAM135B	0	1	1.00	0	1.00E+00
PIK3CA	0	1	1.00	80	1.00E+00
SRGAP3	0	1	1.00	36	1.00E+00
NCOA2	0	1	1.00	84	1.00E+00
ROBO2	0	1	1.00	35	1.00E+00
VAV1	0	1	1.00	30	1.00E+00
EZH2	0	1	1.00	82	1.00E+00
FUBP1	0	1	1.00	93	1.00E+00
KAT7	0	1	1.00	79	1.00E+00
PTPRC	0	1	1.00	30	1.00E+00
LATS1	0	1	1.00	62	1.00E+00
SND1	0	1	1.00	97	1.00E+00
CIC	0	2	1.00	95	1.00E+00
ERBB4	0	1	1.00	42	1.00E+00
LARP4B	0	3	1.00	0	1.00E+00
RAF1	0	1	1.00	91	1.00E+00
HIP1	0	1	1.00	88	1.00E+00
AKT3	0	2	1.00	70	1.00E+00
SALL4	0	3	1.00	31	1.00E+00
NFATC2	0	2	1.00	67	1.00E+00
SETBP1	0	1	1.00	63	1.00E+00
ARHGAP26	0	1	1.00	62	1.00E+00
KIAA1549	0	2	1.00	54	1.00E+00
PDGFRB	0	2	1.00	83	1.00E+00
SMARCE1	0	1	1.00	92	1.00E+00
BCL9L	0	1	1.00	90	1.00E+00
AKT2	0	2	1.00	82	1.00E+00
MALT1	0	1	0.99	57	1.00E+00
CSF1R	0	2	0.99	63	1.00E+00
LMNA	0	2	0.99	84	1.00E+00
DDR2	0	1	0.99	46	1.00E+00
TCF7L2	0	4	0.99	90	1.00E+00
NAB2	0	1	0.99	72	1.00E+00
SGK1	0	1	0.99	49	1.00E+00
HIF1A	0	1	0.98	96	1.00E+00
ARNT	0	1	0.98	89	1.00E+00
HNF1A	0	1	0.97	22	1.00E+00
REL	0	1	0.97	29	1.00E+00
JAK2	0	2	0.97	61	1.00E+00
CCDC6	0	1	0.97	89	1.00E+00

© 2020 Morton SU et al. JAMA Cardiology.

Gene	Total CHD	Total Control	pLI	Heart Expression Rank	Binomial p
MAP3K13	0	3	0.97	36	1.00E+00
QKI	0	2	0.96	97	1.00E+00
PML	0	6	0.94	76	1.00E+00
FAM131B	0	1	0.92	33	1.00E+00
HEY1	0	1	0.92	64	1.00E+00
SEPT5	0	2	0.91	47	1.00E+00
PIM1	0	1	0.89	73	1.00E+00
MYB	0	4	0.88	31	1.00E+00
HLF	0	1	0.88	59	1.00E+00
SIX2	0	1	0.87	22	1.00E+00
TERT	0	1	0.87	36	1.00E+00
CDKN1B	0	2	0.85	91	1.00E+00
FNBP1	0	2	0.85	85	1.00E+00
DAXX	0	2	0.84	69	1.00E+00
BCL11A	0	1	0.83	72	1.00E+00
RAP1GDS1	0	2	0.82	81	1.00E+00
WNK2	0	2	0.79	70	1.00E+00
ZCCHC8	0	2	0.78	85	1.00E+00
FAS	0	2	0.73	26	1.00E+00
ID3	0	1	0.69	88	1.00E+00
RPN1	0	10	0.66	88	1.00E+00
CCR7	0	1	0.65	20	1.00E+00
PAX7	0	1	0.63	12	1.00E+00
LEPROTL1	0	1	0.62	78	1.00E+00
ARAF	0	1	0.62	83	1.00E+00
ELF4	0	1	0.61	84	1.00E+00
FLT3	0	3	0.61	11	1.00E+00
CDX2	0	1	0.55	0	1.00E+00
ACSL3	0	4	0.53	68	1.00E+00
CD28	0	1	0.51	22	1.00E+00
TAL2	0	2	0.49	12	1.00E+00
TLX1	0	1	0.46	20	1.00E+00
CDKN2A	0	3	0.36	11	1.00E+00
SRSF2	0	5	0.35	99	1.00E+00
BCL2	0	1	0.34	63	1.00E+00
RNF43	0	2	0.32	27	1.00E+00
CDH10	0	4	0.31	40	1.00E+00
HIST1H3B	0	1	0.31	32	1.00E+00
AXIN1	0	1	0.25	68	1.00E+00
TCF3	0	1	0.19	89	1.00E+00
RHOH	0	1	0.12	23	1.00E+00
BCL10	0	2	0.12	67	1.00E+00
TPM4	0	3	0.11	95	1.00E+00
TMEM127	0	1	0.10	78	1.00E+00

© 2020 Morton SU et al. JAMA Cardiology.

Gene	Total CHD	Total Control	pLI	Heart Expression Rank	Binomial p
HOXC11	0	2	0.08	0	1.00E+00
NT5C2	0	4	0.05	83	1.00E+00
SDHB	0	1	0.05	98	1.00E+00
ARHGEF10L	0	3	0.04	56	1.00E+00
SETD1B	0	1	0.02	77	1.00E+00
EPHA3	0	1	0.02	44	1.00E+00
NCOA4	0	2	0.01	87	1.00E+00
SH2B3	0	2	0.01	61	1.00E+00
CD274	0	2	0.00	29	1.00E+00
ERC1	0	4	0.00	85	1.00E+00
FOXR1	0	4	0.00	0	1.00E+00
FGFR3	0	1	0.00	39	1.00E+00
OMD	0	2	0.00	27	1.00E+00
FHIT	0	1	0.00	33	1.00E+00
N4BP2	0	7	0.00	57	1.00E+00
PDCD1LG2	0	9	0.00	0	1.00E+00
TFPT	0	2	0.00	66	1.00E+00
HOXA9	0	4	0.00	12	1.00E+00
WT1	0	1	0.00	66	1.00E+00
XPA	0	7	0.00	49	1.00E+00
CIITA	0	5	0.00	27	1.00E+00
SFRP4	0	1	0.00	16	1.00E+00
POLG	0	3	0.00	81	1.00E+00
MYOD1	0	4	0.00	14	1.00E+00
BCL2L12	0	2	0.00	58	1.00E+00
SLC34A2	0	1	0.00	9	1.00E+00
CARS	0	2	0.00	62	1.00E+00
FCRL4	0	2	0.00	0	1.00E+00
PRF1	0	1	0.00	14	1.00E+00
NTHL1	0	2	0.00	42	1.00E+00
DDX10	0	2	0.00	66	1.00E+00
TET2	0	3	0.00	81	1.00E+00

© 2020 Morton SU et al. JAMA Cardiology.

eTable 9. Cohort Subset Analysis

Subset	Number CHD	Number CHD with Risk Var	Number Control	Number Control with Risk Var	Binomial p-value[a]	OR (95% CI)
Overall	4443	642	9808	1099	**2.31E-11**	**1.34 (1.21-1.49)**
European	3106	448	9501	849	**<2.2E-16**	**1.72 (1.52-1.94)**
Non-European	1337	194	307	250	1	0.04 (0.03-0.05)
With OMIM CHD[b] Gene Variant	150	22	64	2	**1.64E-09**	**5.41 (1.23-23.76)**
Without OMIM CHD[b] Gene Variant	4293	576	9744	1080	**1.20E-06**	**1.24 (1.12-1.39)**
With ECA	1482	248	9808	1099	**1.32E-10**	**1.59 (1.37-1.85)**
Without ECA	2340	303	9808	1099	4.75E-03	1.18 (1.03-1.35)
With NDD	1393	209	9808	1099	**9.71E-06**	**1.40 (1.19-1.64)**
Without NDD	950	121	9808	1099	7.61E-02	1.16 (0.95-1.41)
NDD Unknown	1485	221	9808	1099	**9.53E-06**	**1.39 (1.19-1.62)**
ECA + NDD	878	123	9808	1099	6.03E-03	1.29 (1.05-1.58)
Isolated	1379	164	9808	1099	6.94E-01	0.96 (0.80-1.15)
Over 16yo	1105	163	9808	1099	**1.91E-04**	**1.37 (1.15-1.64)**
Under 16yo	3338	479	9808	1099	**1.59E-08**	**1.33 (1.18-1.49)**

[a]Bonferroni significant p-value threshold 3.57E-03
[b]114 OMIM CHD dominant genes; 685 COSMIC genes that are not OMIM CHD genes

© 2020 Morton SU et al. JAMA Cardiology.

eTable 10. Characteristics of genes enriched in CHD patients with congenital anomalies and/or neurodevelopmental delay

Gene*	Total CHD n=4443	CHD+ECA n=1482	CHD+NDD n=1393	Non-CHD n=9808	pLI	HHE	OMIM CHD	Regulatory	Syndrome/ CHD Phenotype	ECA Phenotypes
**KMT2D**	15	10	5	1	1	97	Yes	Yes	Kabuki:<br>LVOTO<br>VSD	Abn facies<br>Microcephaly<br>Low set ears<br>Cleft/arched palate<br>Ectopic kidney,<br>Cryptorchidism,<br>Hip dysplasia<br>Vertebral abn<br>Abnl digits<br>Seizures
**NOTCH1**	12	3	3	0	1	88	Yes	Yes	Adams-Oliver:<br>Aortic valve abn<br>LVOTO<br>Mitral atresia<br>ToF	Airway malacia<br>Low set ears<br>Micrognathia<br>Seizure
**TCF12**	5	2	3	2	0.97	89	No	Yes	Craniosynostosis;<br>ASD, Ebstein,<br>LVOTO	Abdominal heterotaxy<br>Absent corpus callosum<br>AML<br>Bitemporal narrowing<br>Seizures
**NSD1**	4	4	2	1	1	95	Yes	Yes	Sotos:<br>ASD<br>L-TGA<br>VSD	Macrocephaly<br>Hydrocephalus<br>Scoliosis
**CTNNB1**	3	3	2	0	1	99	No	Yes	NDD Disorder:<br>ASD<br>Tricuspid Valve Abn<br>ToF	Astigmatism<br>Hypotonia<br>Microcephaly<br>Micrognathia<br>Scoliosis<br>Strabismus

© 2020 Morton SU et al. JAMA Cardiology.

Gene*	Total CHD N=4443	CHD+ ECA N=1482	CHD+ NDD N=1393	Non-CHD N=9808	pLI	HHE	OMIN CHD	Regulatory	Syndrome/ CHD Phenotype	ECA Phenotypes
**MLLT4**	3	2	-	2	1	92	No	No	None; CAVC, LOVOT, ToF	Arched palate<br>Abdominal heterotaxy
**ARHGEF12**	3	-	3	0	1	91	No	No	None	Hearing loss
**RPL5**	3	2	-	0	0.99	98	Yes	No	Diamond Blackfan Anemia:<br>ASD/VSD<br>LVOTO<br>ToF	Lenticulostriate vasculopathy<br>Macrocephaly
**PTEN**	3	2	2	1	0.98	78	No	No	Cowden:<br>ASD<br>VSD	Abn vertebrae<br>Airway malacia<br>Chiari malformation<br>Hydrocephalus<br>Macrocephaly<br>Scoliosis
**TFRC**	3	2	-	1	0.78	82	No	No	Immunodeficiency:<br>Pulmonary Artery atresia/stenosis,<br>ToF	Frontal bossing<br>Intestinal atresia<br>VATER association
**LPP**	3	-	3	3	0.58	91	No	No	None:<br>LVOTO	Chiari malformation<br>Hydrocephalus<br>Myelodysplasia<br>Neural tube defect

*Genes shown have a nominal burden for LoF variants in CHD + ECA NDD subset. N denotes number of CHD or controls with variants or phenotypes. Abbreviations: Abn, abnormal; ASD, atrial septal defect; CAVC, complete atrioventricular canal; L-TGA, levo-transposition of great arteries; LVOTO, left ventricular outflow tract obstructive lesion; ToF, tetralogy of Fallot; VSD, ventricular septal defect.

© 2020 Morton SU et al. JAMA Cardiology.

eReferences.

1. Gelb, B. *et al.* The Congenital Heart Disease Genetic Network Study. *Circ. Res.* **112**, 698–706 (2013).
2. Opotowsky, A. R. *et al.* Design and Implementation of a Prospective Adult Congenital Heart Disease Biobank. *World J. Pediatr. Congenit. Hear. Surg.* **7**, 734–743 (2016).
3. Krumm, N. *et al.* Excess of rare, inherited truncating mutations in autism. *Nat. Genet.* **47**, 582–8 (2015).
4. Fischbach, G. D. & Lord, C. The Simons Simplex Collection: a resource for identification of autism genetic risk factors. *Neuron* **68**, 192–5 (2010).
5. Genovese, G. *et al.* Increased burden of ultra-rare protein-altering variants among 4,877 individuals with schizophrenia. *Nat. Neurosci.* **19**, 1433–1441 (2016).
6. Sondka, Z. *et al.* The COSMIC Cancer Gene Census: describing genetic dysfunction across all human cancers. *Nat. Rev. Cancer* **18**, 696–705 (2018).
7. Futreal, P. A. *et al.* A census of human cancer genes. *Nat. Rev. Cancer* **4**, 177–183 (2004).
8. McKusick-Nathans Institute of Genetic Medicine, J. H. U. Online Mendelian Inheritance in Man, OMIM®. Available at: https://omim.org/.
9. Homsy, J. *et al.* De novo mutations in congenital heart disease with neurodevelopmental and other congenital anomalies. *Science (80-.).* **350**, 1262–1266 (2015).
10. Jin, S. C. *et al.* Contribution of rare inherited and de novo variants in 2,871 congenital heart disease probands. *Nat. Genet.* **49**, 1593–1601 (2017).
11. Cingolani, P. *et al.* A program for annotating and predicting the effects of single nucleotide polymorphisms, SnpEff. *Fly (Austin).* **6**, 80–92 (2012).
12. Wang, K., Li, M. & Hakonarson, H. ANNOVAR: Functional annotation of genetic variants from high-throughput sequencing data. *Nucleic Acids Res.* **38**, e164 (2010).
13. Karczewski, K. J. *et al.* Variation across 141,456 human exomes and genomes reveals the spectrum of loss-of-function intolerance across human protein-coding genes. *bioRxiv* 531210 (2019). doi:10.1101/531210
14. Mi, H., Muruganujan, A. & Thomas, P. D. PANTHER in 2013: modeling the evolution of gene function, and other gene attributes, in the context of phylogenetic trees. doi:10.1093/nar/gks1118

© 2020 Morton SU et al. JAMA Cardiology.
